# Hexokinase1: A glucose sensor involved in drought stress response and sugar metabolism depending on its kinase activity in strawberry

**DOI:** 10.3389/fpls.2023.1069830

**Published:** 2023-01-27

**Authors:** Runqin Wu, Ximeng Lin, Jinwei He, Ailing Min, Li Pang, Yan Wang, Yuanxiu Lin, Yunting Zhang, Wen He, Mengyao Li, Yong Zhang, Ya Luo, Xiaorong Wang, Haoru Tang, Qing Chen

**Affiliations:** ^1^ College of Horticulture, Sichuan Agricultural University, Chengdu, Sichuan, China; ^2^ Institute of Pomology and Olericulture, Sichuan Agricultural University, Chengdu, Sichuan, China

**Keywords:** hexokinase, glucose signaling, strawberry, drought stress, sugar metabolism

## Abstract

Hexokinase1 (HXK1) is a bifunctional enzyme that plays indispensable roles in plant growth, nitrogen utilization, and stress resistance. However, information on the HXK family members of strawberries and their functions in glucose sensing and metabolic regulation is scarce. In the present study, four HXKs were firstly identified in the genome of *Fragaria vesca* and *F. pentaphylla*. The conserved domains of the HXK1s were confirmed, and a site-directed mutation (S177A) was introduced into the FpHXK1. *FpHXK1*, which shares the highest identity with the *AtHXK1* was able to restore the glucose sensitivity and developmental defects of the Arabidopsis *gin2-1* mutant, but not its kinase-activity-impaired mutant (*FpHXK1^S177A^*). The transcription of *FpHXK1* was dramatically up-regulated under PEG-simulated drought stress conditions. The inhibition of the HXK kinase activity delayed the strawberry plant’s responses to drought stress. Transient overexpression of the *FpHXK1* and its kinase-impaired mutant differentially affected the level of glucose, sucrose, anthocyanins, and total phenols in strawberry fruits. All these results indicated that the FpHXK1, acting as a glucose sensor, was involved in drought stress response and sugar metabolism depending on its kinase activity.

## 1 Introduction

Hexokinase (HXK) is one of the ancient and conserved hexose sensors. It is capable of phosphorylating a variety of hexoses, with glucose being the preferred substrate (Kawai et al., 2005). In the glycolytic pathway, it catalyzes the formation of the glucose 6-phosphate, providing substrates for the oxidative pentose-phosphate, and promoting starch degradation and fat storage. Therefore, HXKs are involved in mediating several important biological processes including seed germination (Moore et al., 2003), seedling growth (Moore et al., 2003), plant vegetative and reproductive development (Rolland et al., 2006), stress responses (Rolland et al., 2006), and senescence (Dai et al., 1999).

Furthermore, HXKs participate in the glucose sensing and signaling process, although the detailed mechanism underlying is still to be elusive. Extensive attention has been paid to how glucose signals are sensed and transmitted to the nucleus through HXKs in recent years (Cho et al., 2006b; Cho et al., 2007; Liu et al., 2022). Due to the absence of a nuclear-targeting motif in the HXK protein, the aid of other molecules is required for the protein translocation among cell compartments (Claeyssen and Rivoal, 2007). In the studies of *Arabidopsis thaliana*, AtHXK1 can form a glucose signaling complex core with the two unconventional partners: Vacuolar proton pump subunit B (VHA-B) and the 19S regulatory particle of proteasome subunit (RPT5B). The complex then binds to the target regulatory protein, which is mainly putative transcription factors, but the downstream target genes are largely unknown (Cho et al., 2006b; Cho et al., 2007). In the most recent study, AtHXK1 interacts with the two catalytic subunits of the Polycomb Repressive Complex 2: CURLY LEAF (CLF) and SWINGER (SWN), facilitating in the histone H3 lysine27 trimethylation and glucose-mediated gene repression (Liu et al., 2022). HXK1 is assumed to locate upstream of secondary metabolism or hormone signaling. The AtHXK1 enhances the degradation of ETHYLENE-INSENSITIVE3 (EIN3) in the nucleus, and inhibits the plant’s response to ethylene when glucose is present (Zhou et al., 1998; Yoo et al., 2009). Moreover, the MdHXK1 phosphorylates Ser-361 of the MdbHLH3 and stabilizes the interaction between MdbHLH3 and MYB1/9/11, thereby promoting anthocyanin biosynthesis at low temperatures (Hu et al., 2016). It can be very interesting to check whether these processes involve the HXK1/VHA-B1/RPT5B or the HXK1/CLF/SWN complex.

The dependence or independence of the sugar kinase activity in the sugar signaling pathway also remains a long-standing question. The intensive mutant screening revealed that the *glucose insensitive2* (*gin2*) mutant of the *AtHXK1* was able to grow on 6% glucose medium, a concentration at which normal seedling growth will be inhibited (Jang et al., 1997). The catalytically inactive mutant of the same protein (G104D and S177A) was found still able to sense this sugar signal, implying that the glucose sensing was uncoupled from the catalytic activity (Moore et al., 2003). Studies of the rice HXK5 and HXK6 further support this conclusion (Cho et al., 2009). In contrast, the OsHXK7 depends on its catalytic activity to rescue the sugar sensing and signaling defects of the *gin2-1* (Kim et al., 2016). KnHXK1, the only HXK coding protein found in the charophyte alga genome also relies on its sugar-phosphorating activity to restore glucose signaling (Ulfstedt et al., 2018). More evidence is needed to explore the common regulatory routes behind these contrasting results.

Strawberries are prized by consumers for their enjoyable aroma and flavor profiles, and are economically significant worldwide. *FaHXK1* has been reported to be highly expressed in high-sugar content cultivars (Lee et al., 2018). Additionally, the external application of microelements Boron (B) and Zinc (Zn) affects the expression of the *FaHXK1* in strawberry leaves and fruits, which may influence the fruit count per inflorescence and the individual fruit weight (Yulia et al., 2016). In production, the plant is sensitive to drought stress, requiring regular irrigation to produce optimum yields. Sugar signaling and transport are believed to play critical roles in the plant’s responses to drought stress (Kaur et al., 2021). *F. pentaphylla* (commonly named quinquefoliolate strawberry) is one of the four wild *Fragaria* species in China. It is mainly distributed at an altitude of 1034 to 3035 m from 29°31’-33°22’N to 101°57’-108°13’E in Sichuan, Gansu, and Shaanxi Province (Li et al., 2014). It has red and white fruit with a strong fragrance. Its red fruit is rich in anthocyanin and other beneficial flavonoids due to the higher expression of flavonoid biosynthesis genes than *F. nilgerrensis* (Shen et al., 2020). Meanwhile, resources screening indicated that the plants are with outstanding resistance to various biotic stress and abiotic stress including drought (Xue et al., 2005). The high-quality genome sequence of the *F. pentaphylla* was released recently (Qiao et al., 2021; Sun et al., 2022), which makes it an ideal endemic resource for strawberry breeding.

In this study, we sought to explore whether the strawberry HXK1 participates in drought responses, and is the kinase activity of the protein is required if so. Based on the currently available strawberry genome resources, four *FvHXKs* and *FpHXKs* in *F. vesca* and *F. pentaphylla* have been identified and characterized. Meanwhile, a catalytically impaired mutation was introduced (FpHXK1^S177A^). The sugar-signaling function of the FpHXK1 was confirmed. The contribution of both FpHXK1 and FpHXK1^S177A^ to the drought stress responses and strawberry fruit flavor was also investigated.

## 2 Materials and methods

### 2.1 Plant materials and growth conditions

Wild *F. pentaphylla* plants (white fruit type) were collected in Guangyuan City of Sichuan Province in 2017. They were propagated vegetatively through stolones in a local greenhouse under natural climatic conditions. Different types of tissues including the roots, crowns, young and mature leaves were collected from at least three plants and pooled for one biological sample. Three replicates were done in this study.

For gene cloning, healthy young leaves of the *F. pentaphylla* were used for total RNA extraction. Seeds of the *gin2-1* (SALK_034233C) were obtained from the Fuzhou AraShare Biotechnology company. The T-DNA insertion was confirmed using reverse transcription PCR (RT-PCR) methods with the flanking primers as suggested (http://signal.salk.edu/ ) (**Supplementary Table 1**).

Seeds were stratified for 72 hours at 4°C before being sowed in soil and transferred to a growth chamber (16 h: 8 h, 150 μmol^−1^ m^−2^ s^−1^, 22°C). In the glucose-repression assays, seedlings were grown on a liquid medium (1/2 Murashige-Skoog, 0.1% agar powder, and 6% glucose) under the 16-h-light/8-h-dark photoperiod for six days. For the low-nutrient and low-light assay, seedlings were grown on 0.2% glucose and 1/10 MS medium for eight days under constant dim light (15 μmol^−1^·m^−2^·s^−1^) condition.

### 2.2 Data mining of the strawberry hexokinase genes

The *F. vesca* genome file (v4.0.a2) was downloaded from the GDR database (https://rosaceae.org ). The most recently released *F. pentaphylla* (v1.0) genome was also used. The Hidden Markov Model (HMM) profile of the HXK family domain: PF00349 or PF03727 was used as a query in an HMM search (*p*< 0.001). The NCBI conserved domain database (CDD, accessed on 20^th^ September, 2021) was used to check all candidate proteins for the presence of the conserved HXK domain. Those proteins with incomplete HXK domains were removed. The protein molecular weight (MW) and the protein isoelectric point (pI) for the putative HXK proteins were calculated *via* ExPASy (http://web.expasy.org/protparam ). Transmembrane helices were predicted using TMHMM (v2.0). The nuclear localization signal (NLS) of FpHXK1 was predicted at predictNLS (https://predictprotein.org/ ). The conserved amino acid residues corresponding to the adenosine triphosphate binding and phosphoryl transfer sites were identified as described by Karve et al. (2008).

### 2.3 Sequence alignment and phylogenetic tree reconstruction

Putative HXK family members of other species were also isolated as described above. Genome sequences of ten plants including *A. thaliana* (Araport11), apple (*Malus domestica*, v1.1), peach (*Prunus persica*, v2.1), grape (*Vitis vinifera*, v2.1), tomato (*Solanum lycopersicum*, ITAG4.0), potato (*Solanum tuberosum*, v6.1), rice (*Oryza sativa*, v7.0), moss (*Physcomitrium patens*, v3), the unicellular green algae (*Chlamydomonas reinhardtii*, v5.5), and cyanobacteria (*Spirodela polyrhiza*, v2) were obtained from the Phytozome database. The tobacco (*Nicotiana benthamiana*, NbD) HXKs and the HXK1/HXK2 of yeast (*Saccharomyces cerevisiae*) were obtained from the database of NCBI. HXK3s of *Prunus cerasifera* and *P. munsoniana* were retrieved from the literature of Pérez-Díaz et al. (2021). The proteome file of *Caenorhabditis elegans* (WS286) was obtained from the WormBase (https://wormbase.org/ ). HK1-4 of *Homo sapiens* were retrieved from the UniProt collection. A multiple sequence alignment was performed using the MAFFT software implemented in the Phylosuite (v1.2.2) package with default parameters. A phylogenetic tree was reconstructed using a maximal likelihood method in the IQ-Tree package following the JTT+I+G4 substitution model, with 10000 fast-bootstrap replications. The online tool iTOL (https://itol.embl.de/upload.cgi ) was employed to visualize the phylogenetic tree.

### 2.4 Gene structure and conserved motif analysis

The exon-intron structure of HXK genes was displayed in the TBtools software (Chen et al., 2020). The Multiple Expectation maximizations for Motif Elicitation (MEME) tool (Bailey et al., 2009) was used to estimate conserved motifs with default parameters.

### 2.5 Cloning of the FpHXK1 and catalytically inactive mutation introduction

A cetyltrimethylammonium bromide (CTAB) based method was used to isolate total RNA from young leaves of *F. pentaphylla*. First-strand cDNAs were synthesized from one microgram of total RNA with random primers, following the instructions of the ABScript II RT Mix for qPCR with gDNA Remover kit (ABclonal, Wuhan, China). The full-length coding sequence (CDS) of the *FpHXK1* gene was cloned by RT-PCR using gene-specific primers for gene cloning: FpHXK1-Fwd and FpHXK1-Rev (**Supplementary Table 1**).

Based on the sequence alignment results and the previous findings of Moore et al (2003) and Hu et al. (2016), the 177^th^ serine of FpHXK1 was mutated to alanine (S177A), expecting to impair the catalytic activity of the protein. To this end, SOE-PCR (Tan et al., 2013) was applied using primers FpHXK1^S177A^-Fwd and FpHXK1^S177A^-Rev (**Supplementary Table 1**). The amplicons of *FpHXK1* and *FpHXK1^S177A^* were inserted into the pCambia1301 vector before sequencing confirmation.

### 2.6 Recombinant protein production of FpHXK1/FpHXK1^S177A^ in *N. benthamiana*

The constructs carrying the *FpHXK1* or *FpHXK1^S177A^* infusion with 6×His tag were transiently expressed in *N. benthamiana* leaves using the pEAQ-HT system (Sainsbury et al., 2009). Five days after infiltration, the infiltrated leaf tissues were collected and homogenized in three volumes of protein extraction buffer (250 mM Tris-HCl pH 8.0, 150 mM NaCl, 1% [v/v] Triton X-100, 0.1% [v/v] protease inhibitor cocktail (Beyotime, Shanghai, China), 2% (v/v) β-mercaptoethanol). The protein concentration was determined by using the Bradford assay (Beyotime, Shanghai, China) after centrifugation. The total protein was used for the subsequent kinase activity test. The plants infiltrated with the pEAQ-HT empty vectors served as a negative control.

### 2.7 Kinase activity assay for FpHXK1/FpHXK1^S177A^

The HXK kinase activity in the crude extract was determined using a commercial kit (Sinobestbio, Shanghai, China). HXK catalyzes the synthesis of glucose 6-phosphate (G6P) from glucose. G6P is further dehydrogenated to form NADPH, which has a characteristic absorption peak at 340 nm. The changes in the absorbance after adding the substrates were monitored for five minutes at 25°C. An increase of 0.01 per minute at OD_340_ (production of 1 nmol NADPH) is defined as one unit of catalytic activity (U) (Fox et al., 1998). The enzyme activity was expressed as U·mg^−1^ protein by the formula:


$$\text{kinase }(\text{U}\cdot {\text{mg}}^{-1}\text{ protein})=\frac{\Delta \text{A}\times {\text{V}}_{\text{total}}\times 10^{9}}{\epsilon \times \text{d}\times {\text{V}}_{\text{sample}}\times {\text{C}}_{\text{protein}}\times \text{T}}$$

where ΔA indicates the changes in absorbance at 340 nm, V_total_ indicates the total reaction volume, *ϵ* is the molar extinction coefficient for NADPH at 340 nm (6.22×103 L·mol^−1^·cm^−1^), d: Light path length of the cuvette (1 cm), V_sample_ indicates the sample volume added to the reaction, C_protein_: the protein concentration in the sample (mg·ml^−1^), T indicates the reaction time (5 min).

The recombinant proteins represented by the 6×His epitope were firstly normalized against the internal actin protein using western blot analysis. The background endogenous HXK activity in the plants with the empty vector control was then subtracted from the HXK activity in the FpHXK1 and FpHXK1^S177A^ infiltrated plants. A total of 40 μg of extracts were separated on 10% SDS-PAGE gels. In the western blot assay, separated proteins were transferred to polyvinylidene difluoride membranes and probed with the anti-His-Tag rabbit monoclonal antibody (PTM Biolab, Chicago, IL, USA) or the anti-plant-Actin mouse monoclonal antibody (Abbikne, Wuhan, China). Anti-rabbit or anti-mouse horseradish peroxidase (HRP)-linked secondary antibodies were then used. Signals were generated by using the BeyoECL Moon chemiluminescence kit (Beyotime, Shanghai, China) and captured by the GelView 6000Plus system (BLT, Guangzhou, China).

### 2.8 Complementation of the *A. thaliana gin2-1* mutant

The plants of *gin2-1* were planted in 10 × 10 cm pots with substrate matrix and transformed by the floral dip method (Zhang et al., 2006) using *Agrobacterium tumefaciens* strain GV3101. Hygromycin B (15 mg·L^−1^) was used to select transgenic plants. The resistant plants were further confirmed by checking the integration of the T-DNA insert using PCR with primers listed in the **Supplementary Table 1**. Stable T3 generation plants were used for sugar sensitivity and drought resistance tests.

### 2.9 Plant stress treatment

Polyethylene glycol 6000 (PEG-6000) was used to mimic drought stress conditions. The growth change of *Arabidopsis* seedlings under this stress condition was tested referring to the method of Verslues et al. (2006). Briefly, PEG-6000 dissolved into a buffer solution (1/2 MS and 6 mM MES) to a final concentration of 15% was overlaid on the prepared agar plates (v:v=3:2) for 12 hours. The wild type, *gin2-1*, *35S::FpHXK1*, and *35S::FpHXK1^S177A^* seeds were plated on these PEG-infused plates (1/2MS, 1.5% agar powder, 0.5% sucrose, 15% PEG-6000). They were stratified for 72 h at 4°C, and transferred to a growth chamber (16 h:8 h, 150 μmol m^–2^ s^–1^, 22°C) for 10 days. Strawberry seedlings were cultured on MS medium containing 3% sucrose for three months under a daily cycle of 16 h light and 8 h dark at 22°C. Seedlings with roots were carefully transferred out. The roots were washed with running water to get rid of the growth medium. Then the seedlings were hydroponically cultured in 20% PEG-6000 solutions at 40°C, and sampled 0, 3, 6, 12, 24 and 48 h after treatment.

### 2.10 Determination of the main antioxidant enzyme activity and the physiological responses

We measured the activities of superoxide dismutase (SOD) and catalase (CAT), and the accumulation of malondialdehyde (MDA) and proline (Pro) of the samples at different intervals after treatment. Briefly, the whole strawberry plantlets with different treatments were ground in liquid nitrogen. Sample powders (0.1 g) were extracted using 1.0 mL of K_2_HPO_4_/KH_2_PO_4_ buffer (pH7.8) at 4°C for 30 min. After centrifugation at 8000 g for 10 min at 4°C, the supernatant was collected for testing. The CAT activity was tested by monitoring the depletion of H_2_O_2_ per gram fresh tissue per minute within 5 min at 240 nm (BC0200, Solarbio, Beijing, China). The SOD activity was determined after the methods of Cheng et al. (2015). The riboflavin, nitroblue tetrazolium (NBT), methionine, and ethylenediamine tetra-acetic sodium were sequentially added to 100 μL extract to a final concentration of 2 μM, 65 μM, 13 μM, and 1 μM, respectively, in the same buffer. The solution was transferred to a glass tube and illuminated (4000 Lux) at 25°C for 20 min. The absorbance of blue formazan was measured at 560 nm. The amount of protein that caused the 50% NBT reduction was defined as one unit of SOD activity. To determine the MDA content, thiobarbituric acid (TBA) was added to 100 μL sample solution and incubated at 95°C for 30 min. The mixture was cooled on ice, and spun at 10000 g for 10 min at 25°C. The reaction of the two compounds forms a red product which could represent the amount of MDA. The difference between the absorbance values at 532 nm and 600 nm was recorded to calculate the concentration. The A107-1-1 kit for proline quantification was used (Jiancheng Bio, Nanjing, China). After mixing 2 ml of the working solution included in the kit with 0.5 ml of the sample supernatant in a glass tube, the mixture was boiled for 30 min, and the reaction was terminated on ice. The absorbance was measured at 520 nm. A serial concentration of L-proline was used to draw a standard curve, with which the final content of Pro in the samples was determined. All absorbance were measured using a Varioskan LUX microplate reader (Thermo, Waltham, MA, USA). Three biological repeats and three technical replicates were carried out for all the physiological tests.

### 2.11 Determination of the ABA content in the stress treated strawberry plantlets

We detected the changes of ABA level throughout 0-48 h treatment duration using an ABA enzyme-linked immunoassay kit (Gersion, Beijing, China). One gram of sample powder was extracted with 9 ml phosphate buffer saline (PBS, PH 7.5) for 30 min, and then centrifuged at 16000 g for 15 min. Afterwards, the supernatant was used for the determination of ABA content according to the manufacturer’s protocol based on a competitive enzyme immunoassay technique. Fifty microliters of the diluted standard included in the kit or samples were added into the plate wells which were pre-coated with antibody specific to ABA. After incubation with 100 μL of HRP-conjugated secondary antibody at 37°C for 60 min, five washes were done with washing buffer in the kit. Fifty microliters of substrate solution A and B were added respectively. The mixture was incubated for 15 min at 37°C. After the reaction was stopped with 50 μL stop solution, the absorbance spectrum was read at 450 nm using a Varioskan LUX Multimode Microplate Reader (Thermo, Waltham, MA, USA) within 15 min.

### 2.12 Transient overexpression of FpHXK1/FpHXK1^S177A^ in strawberry fruits

Following the protocol of Zhao et al. (2019), *FpHXK1* as well as its catalytically impaired mutant *FpHXK1^S177A^* were transiently expressed in strawberry fruits. Fruits at the large green stage were collected. For each construct (*pCambia1301*, *35S::FpHXK1*, *35S::FpHXK1^S177A^*), at least 30 fruits were used. The suspended agrobacterium GV3101 cells were infiltrated at OD_600_ = 0.5 diluted in the infection buffer (10 mM MgCl_2_, 200 μM Acetosyringone, and 10 mM MES, pH 5.6). The fruits were incubated overnight in the dark after injection. The next day, the culture was returned to light culture conditions in a growth chamber (23°C, 16 h light/8 h dark, RH: 90%) until the entire biological replicate was ready to be collected. As a reference, we also employed an empty vector carrying the *mGFP* gene to check the infection efficiency. It also serves as a guide for sample collecting. In the current experiment, the infiltrated samples were collected seven days after infiltration when the highest expression of the target gene was observed. Three biological repeats were conducted for each transient assay.

### 2.13 Determination of the sugars, anthocyanins, and total phenols of the strawberry fruits

The glucose, fructose, sucrose, citric acid, and the total anthocyanin content were determined following our previous study (Chen et al., 2022). To determine the total phenols content, a folin-ciocalteu method was used. Lyophilized strawberry tissues (1 g) were homogenized in 3 mL 80% (v/v) acetone, and then incubated for 1 h at room temperature. The homogenate was centrifuged at 4500 g for 10 min at 4°C. Fifty microliters of the supernatant were mixed with 1 ml of 2% Na_2_CO_3_. After 5 min of reaction, 50 µL of 50% Folin-Ciocalteu’s phenol reagent was added. The absorbance at 655 nm was measured after an incubation at 20°C for 30 min in the dark. The total phenol content was calculated based on the standard curve of absorbance value by the known concentration of gallic acid. To quantify the total flavone content, samples were pretreated the same as for the total phenol testing. A volume of 120 µL extracts was mixed with 360 µL of 90% ethanol, 24 µL 2% AlCl_3_, 24 µL 1 M KAC, and 672 µL distilled water. After an incubation at room temperature for 40 min in the dark, the absorbance at 415 nm was measured. Quercetin was used as equivalent standards in the test.

### 2.14 Relative gene expression analysis

Gene expression levels of the target genes were measured using RT-qPCR. Briefly, diluted cDNA (10×) was used as templates. Amplification products were monitored using SYBR-Green I (TaKaRa, Dalian, China) in a CFX Connect™ Real-Time System (Bio-Rad, Hercules, CA, USA). The relative expression data was analyzed using the 2^−ΔΔCt^ method. For each biological sample, three technical replicates were analyzed. The *TUBULIN3* (Barbier et al., 2021) and *FaActin* gene (Chen et al., 2022) were used for normalization in Arabidopsis and strawberry, respectively. Primers of FpHXK1 and FpHXK1^S177A^ used for RT-qPCR in this study were listed in **Supplementary Table 1**. All reactions were conducted in a 20 μL system, containing 10 µL 2×SYBR Green fast qPCR mixture, 0.5 µmol forward primer and reverse primer, 1 μL cDNA. A two-step PCR cycling method was followed: one cycle of pre-denaturation at 95°C for 3 min, flowed by 45 cycles of 95°C for 5 s and 60°C for 30 s.

When the expression of the photosynthetic and stress-responsive genes was analyzed, semiquantitative RT-PCR was adopted following the method of Cho et al. (2006b). In *Arabidopsis*, the *carbonic anhydrase* (*CAA*) and *chlorophyll a/b binding protein* (*CAB*) gene were selected. The *AtUBQ10* was used as a reference. In strawberry, drought responsive *RD29A, DREB2A* and *PP2C* genes (Li et al., 2021) were selected. Primers were listed in **Supplementary Table 1**. The *FpActin* was used as a reference.

### 2.15 Statistical analysis

The data were expressed as means ± standard error of means calculated in the SPSS software (v27.0, IBM, Armonk, NY, USA). Tukey’s multiple comparison test was applied when exploring differences among means in the same treatment. A value of *p<* 0.05 was considered statistically significant. ImageJ (v1.52a) was used to quantify the relative amount of protein bands of FpHXK1 and FpHXK1^S177A^ in the western blot films.

## 3 Results

### 3.1 Identification and characterization of the hexokinase genes in strawberries

**Figure 1:**
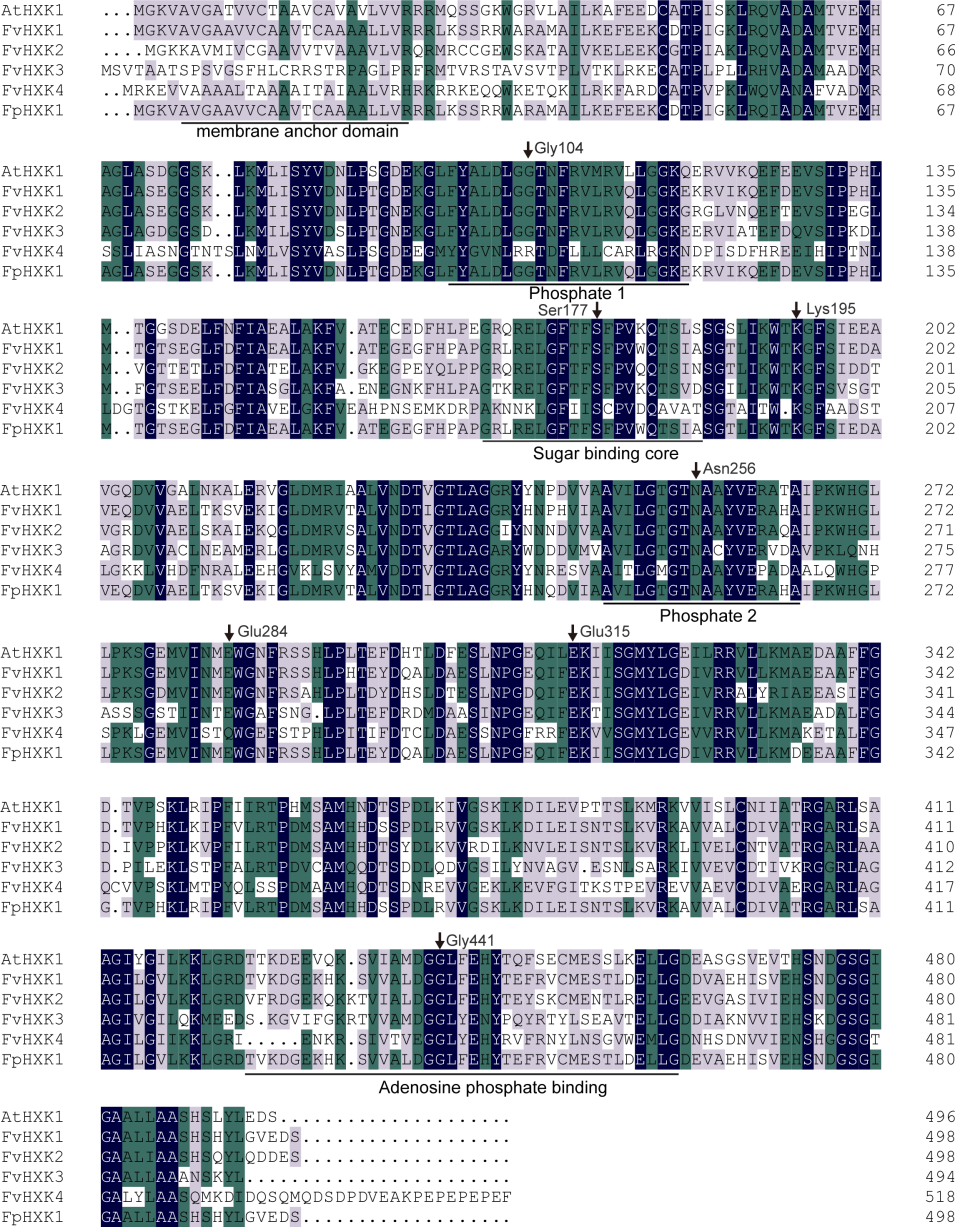
Amino acid alignment analysis of five identified strawberry HXKs with AtHXK1. Regions with known functions were underlined. The black arrows represented conserved amino acids (counting based on AtHXK1), which are responsible for the catalytic activity and glucose and adenosine bonding referring to Karve et al. (2008). The shadings in black, green, and light purple reflect 100%, 75%, and 50% amino acid residue conservation.

With the available high-quality complete genome sequences of and *F. vesca* (Li et al., 2019) and *F. pentaphylla* (Sun et al., 2022), the HXK coding genes were firstly screened in the genome. After removing those with incomplete HXK domain, we finally obtained four *FvHXKs* and four *FpHXKs* (**Table 1**). They shared high sequence identity to that of *AtHXK1*. The deduced protein length ranged from 494 (FvHXK3) to 562 (FpHXK3), with the molecular weight (MW) varying from 53.02 to 60.99 kDa. The isoelectric point (pI) varied from 5.25 (FvHXK3) to 6.15 (FvHXK1) (**Table 1**). One transmembrane domain was predicted to be exist within the first 50 amino acids in all HXKs except HXK3s, suggesting their subcellular association with the membranes within the cell. The FvHXK3/FpHXK3 protein does not have such characteristics (**Supplementary Figure 1**). Multiple sequence alignments of the isolated HXKs allowed the identification of several functional conserved domain and residuals as shown in **Figure 1**. The glucose-binding residues of AtHXK1, FvHXK1/FpHXK1, FvHXK2/FpHXK2, and FvHXK3/FpHXK3 were identical. In the FvHXK4/FpHXK4, alternative residuals were found in the known critical sites (arrow heads in **Figure 1**): Arg107, Asp261, and Gln289, which may lead to its compromised function in the glucose binding. In addition, FvHXK4/FpHXK4 has an extra-long amino acid sequence ‘MQDSDPDVEAKPEPEPEPEF’ in the C-terminal. Therefore, we assume that FvHXK4/FpHXK4 is a hexokinase-like protein with different functions from other HXKs. Complete alignment of *F. pentaphylla* HXKs was presented in **Supplementary Figure 2**.

**Table 1:** Basic information of hexokinase genes in *F. vesca* and *F. pentaphylla*.

Name	Transcript ID	CDS length (bp)	Protein			
			Length (a.a.)	pI	MW (kDa)	Transmembrane domain position
FvHXK1	FvH4_1g06270.t1	1497	498	6.15	53.97	5-24
FvHXK2	FvH4_6g32470.t2	1497	498	5.95	54.10	5-23
FvHXK3	FvH4_2g38530.t1	1485	494	5.25	53.02	–
FvHXK4	FvH4_1g18730.t1	1557	518	6.01	56.81	5-24
FpHXK1	Fpe07G005790.1	1497	498	6.00	53.99	5-24
FpHXK2	Fpe01G031800.1	1497	498	5.84	54.07	5-23
FpHXK3	Fpe04G039540.1	1689	562	5.66	60.99	–
FpHXK4	Fpe07G018400.1	1557	518	6.01	56.92	5-24

CDS, coding sequence; pI, protein isoelectric point; MW, the molecular weight of the proteins.

### 3.2 Phylogenetic relationship of the hexokinase genes across species

**Figure 2:**
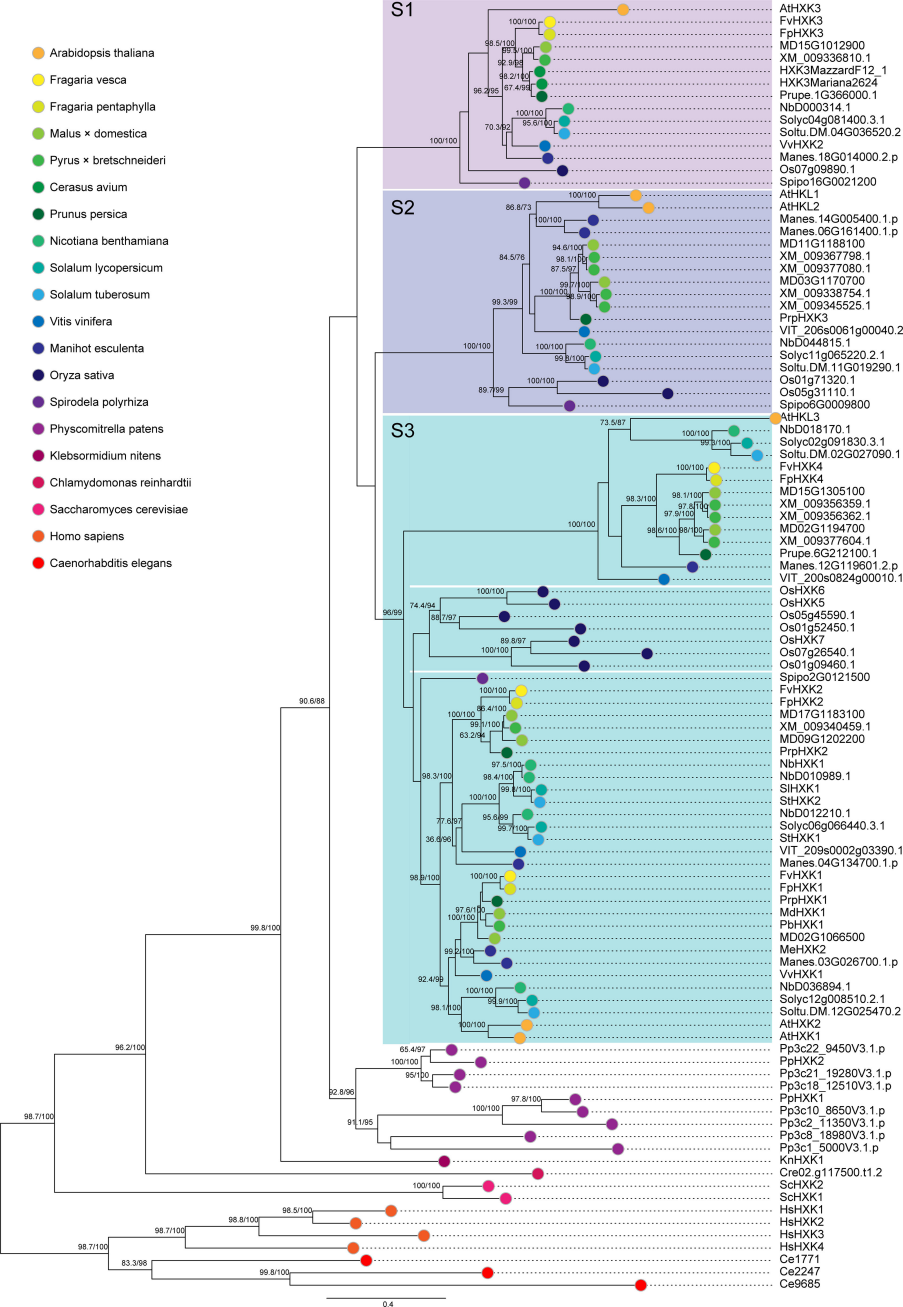
The maximum likelihood phylogenetic tree based on the identified HXK proteins. The phylogenetic tree was generated based on the JTT+I+G4 substitution model with 10000 fast bootstrap replicates. The accession number of the proteins could be found in the **Supplementary Table 2**.

Using the same strategy, we identified a total of 104 HXKs in 20 species (**Supplementary Table 2**), covering a wide spectrum of green plants (Viridiplantae), from green algae (*Klebsormidium nitens*) to woody land plants. We also included two Animalia species, *C. elegans* and *H. sapiens* as outgroups. Only one HXK gene was found in the green algae genome (*K. nitens* and *C. reinhardtii*), representing the ancestral form of HXK genes (**Figure 2**). In contrast, nine HXKs were encoded in the moss (*P. patens*) genome. This obvious expansion of the gene highlighted the essential role of HXKs or sugar metabolism and signaling in the process of land colonization. The HXKs in land plants, including both dicots and monocots were clustered into three large subclades (S1 to S3) with high credibility (**Figure 2**). The subclade S1 contains one HXK member from each tested species, while in the S2 subclade, two HXK proteins are included for most species. Noteworthy, strawberry does not have members belonging to this S2 group. The third subclade (S3) seems to be more complex, which is further diverged into two groups. The first one consists of one hexokinase-like protein from each species exclusively belongs to the angiosperm dicots. The second one comprises seven HXKs of rice, the only monocots species included in this study, and two HXK members from each of the rest dicotyledonous plants. It was very interesting to observe that only three HXK coding genes were present in the genome of *S. polyrhiza*. These three proteins evenly fell into the observed three subclades. These results indicated that the HXK genes were subjected to various expansion/contraction in different species, which might lead to different functions so as to accommodate the requirement of ever-changing environment.

### 3.3 Gene structure and conserved protein motifs of hexokinases in diploid strawberries

**Figure 3:**
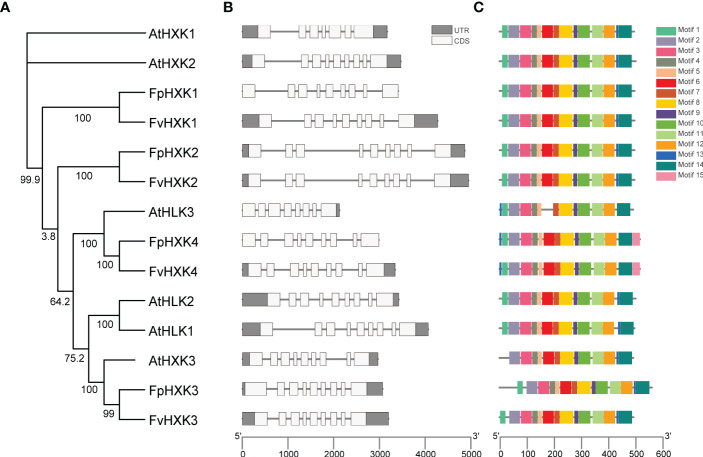
Protein motifs and gene structures of the HXKs in *Arabidopsis* and strawberry. **(A)** The phylogenetic dendrogram of the hexokinase genes in the *Arabidopsis* and strawberry genome. **(B)** The exon/intron structures of the HXK genes. The relative position and size of the exon (white box) and UTR (gray box) can be estimated using the scale bar at the bottom. **(C)** The putative conserved motifs in HXKs. Different motifs and their relative positions are represented by the colored boxes at the top right.

Based on the gene annotation profile, the gene structure of the identified HXKs in *F. vesca* and *F. pentaphylla* were compared (**Figure 3**). Relative conservation was observed for both species. All HXK genes consist of eight introns and nine exons, despite that the intron length varies among genes. This feature was similar to that was observed in *Arabidopsis* (Karve et al., 2008). The known function of the first conserved motif is elusive. The motif 2 to motif 14 were identified in all HXK proteins (**Figure 3B**) except AtHKL3, which lacks the sixth motif structure. The second to seventh motif corresponds to the Hexokinase domain_1 structure, while the motif 10 to motif 14 corresponds to the Hexokinase domain_2. The 8^th^ motif spans these two domains (**Supplementary Table 3**). The 15^th^ motif, annotated as the Trypan_PARP (PF05887) domain, was only found in FpHXK4 and FvHXK4. All these results indicated that the *HXK* genes and proteins were highly conserved, reflecting their functional significance in the evolutionary history.

### 3.4 Hexokinase activities of the FpHXK1 and the mutant FpHXK1^S177A^

Previous studies have shown that the glucose signaling function of hexokinase is dependent or independent on its catalytic activity (Cho et al., 2009; Moore et al., 2003; Ulfstedt et al., 2018; Kim et al., 2016). In order to explore the relationship between the sugar signaling function and the catalytic activity of HXK in strawberry, the *FpHXK1* gene was cloned from *F. pentaphylla* using gene specific primers (**Supplementary** **Table 1**). *FpHXK1* contains an open reading frame (ORF) with a length of 1,497 bp, coding for 498 amino acids. Twenty-five SNPs were found when compared with the *FvHXK1* gene, leading to eight different amino acids.

**Figure 4:**
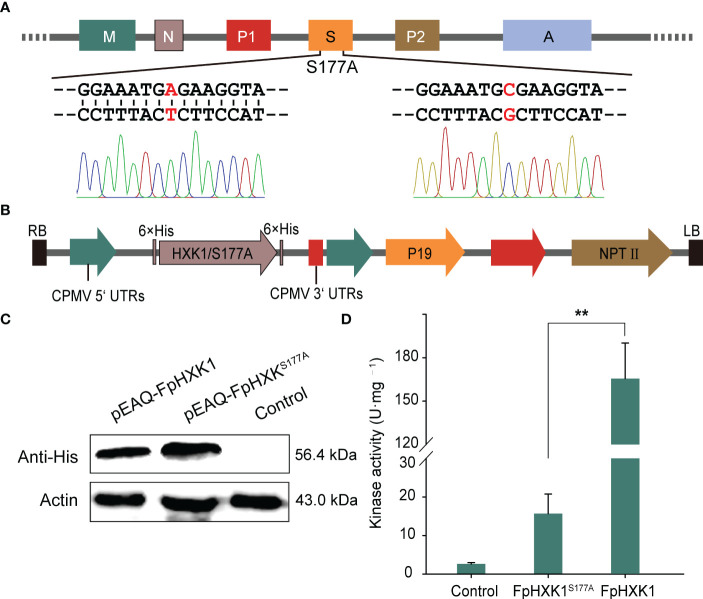
Site-directed mutagenesis of *FpHXK1* and its impact on the kinase activity. **(A)** Schematic representation of *FpHXK1* and its catalytically inactive mutation site S177A. Mitochondrial targeting signals and NLS are indicated as M and N rectangles. P1 and P2 indicate the conserved phosphate regions 1, and 2. The region S and A indicate the conserved sugar-binding and adenosine-binding domains, respectively. **(B)** Diagrammatic representation of the T-DNA region of pEAQ-HT (His tag). Black boxes, T-DNA borders; green arrows, promoter sequences; and red arrows, terminator sequences. **(C)** Western blot analysis of the recombinant protein of FpHXK1 and FpHXK1^S177A^ in *N. benthamiana*. **(D)** Kinase activity assay of the extracts of recombinant FpHXK1 and FpHXK1^S177A^. ** indicates significant difference at the level of *p*< 0.01.

The amino acid sequence alignment revealed that the Serine at position 177 was conserved (**Figure 1**). Since the structure model of the AtHXK1 and AtHXK1^S177A^ shared the same spatial conformation in the sugar binding motifs (Feng et al., 2015), we directly used targeted mutagenesis (**Figure 4A**) to test the critical role of the corresponding serine of FpHXK1 in the enzyme’s catalytic activity. The wild-type gene and the mutant were ligated into a binary vector of pEAQ-HT (**Figure 4B**), and transiently expressed in *N. benthamiana* using an agroinfiltration method. The western blot results (**Figure 4C**) indicated the successful production of the recombinant protein of FpHXK1 and FpHXK1^S177A^. Kinase-activity tests using the extracted proteins demonstrated that the introduced mutation had dramatically reduced (>90%) the sugar-phosphorating ability of the protein (**Figure 4D**), although residual kinase activity still exists in the mutation. These results confirmed that the serine residual of the FpHXK1 was one of the determinants of catalytic activity, which could be used to explore the possibility of uncoupling the signaling function from the metabolic reaction.

### 3.5 FpHXK1 serves as a glucose sensor depending on its catalytic activity

To further investigate FpHXK1’s role in glucose sensing and signaling, complementation of the Arabidopsis *gin2-1* mutant (SALK_034233C) with the *FpHXK1* as well as the *FpHXK1^S177A^* was carried out. The T-DNA insertion of *gin2-1* was confirmed first as shown in **Supplementary Figure 3.** Transgenic plants were selected on hygromycin-containing medium and validated through PCR. Lines with higher levels of expression were used in the subsequent analyses.

**Figure 5:**
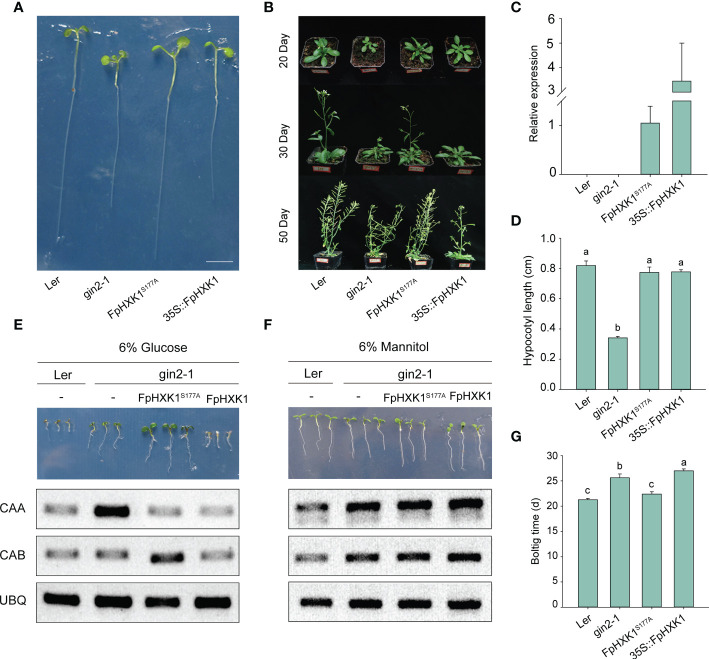
Complementation of (*A*) *thaliana* mutant *gin2-1* with *FpHXK1* or *FpHXK1^S177A^*. **(A)** Seedling-growth analysis of *Ler, gin2-1, 35S::FpHXK1*, and *35S::FpHXK1^S177A^* under low-light and low-nutrient conditions (0.2% Glc, 1/10 MS) for eight days. Scale bar, 0.5 cm. **(B)** Plant phenotype of *Ler, gin2-1*, and complementation of *gin2-1* with *35S::FpHXK1*, and *35S::FpHXK1^S177A^*. **(C)** The relative gene expression level of *FpHXK1* and *FpHXK1^S177A^* in the *gin2-1* background. *TUB3* was used as an internal control. In the *Ler* wild-type and *gin2-1* plants, no *FpHXK1* or *FpHXK1^S177A^* expression could be detected. **(D)** Hypocotyl length of seedings under low-light and low-nutrient. Values were presented as mean ± SEM of 6-10 seedings. Statistically significant differences among samples were tested by Tukey’s test, significantly different (p< 0.05) were indicated by different letters, as determined by a two-way analysis of variance. **(E)** Seedling-growth phenotype and the expression level of the *CAA* and *CAB* gene in *Ler*, *gin2-1, 35S::FpHXK1*, and *35S::FpHXK1^S177A^* lines in 1/2 MS liquid medium with 6% glucose. Scale bar, 0.5 cm. **(F)** Seedling-growth phenotype and the expression level of the *CAA* and *CAB* gene in *Ler*, *gin2-1, 35S::FpHXK1*, and *35S::FpHXK1^S177A^* lines in 1/2 MS liquid medium with 6% mannitol. Scale bar, 0.5 cm. **(G)** Bolting time of the *gin2-1*, *Ler* wild-type and complementation lines.

Compared to the *gin2-1*, the *35S::FpHXK1* and *35S::FpHXK1^S177A^* complementation seedlings exhibited normal growth under low-light and low-nutrient conditions (**Figures 5A–D**). In contrast, the growth of the *35S::FpHXK1* transgenic seedlings was suppressed, resembling the *Ler* wild-type on 1/2 MS medium with 6% glucose (**Figure 5E**). The *35S::FpHXK1^S177A^* complementation seedlings, however, did not respond to high-glucose repression, with normal green cotyledons. It exhibited vigorous growth, even better than those of the *gin2-1* seedlings (**Figure 5E** and **Supplementary Figure 4**). The photosynthesis-related genes *CAA* and *CAB* were reported to be regulated downstream of the HXK-mediated sugar signaling (Cho et al., 2006b). In the wild-type, both genes were significantly suppressed under high glucose conditions. This repression was largely released for the *CAA* gene in the *gin2-1* but retained in plants complemented with *FpHXK1^S177A^* and *FpHXK1*. In contrast, the inhibition of the CAB gene was abolished through complementation with *35S::FpHXK1^S177A^* but maintained in those with *35S::FpHXK1* (**Figure 5E**). All these results were not observed in plants grown in medium containing 6% mannitol (**Figure 5F**), indicating that the changes were not ascribed to osmotic stress.

For adult plants, the *gin2-1* mutant exhibits typical growth defects (**Figure 5B**) as described previously (Moore et al., 2003). The *35S::FpHXK1^S177A^* transgenic plants also showed growth deformities. Its bolting stage, flowering stage, the slender branching appearance as well as the number of rosette branches were similar to the *gin2-1* plants. It was also characterized by the shrinking leaves. When complementation with *FpHXK1*, the plants displayed delayed but normal growth like those of wild-type plants (**Figures 5B, G** and **Supplementary Figure 5**). All these results provided compelling evidence that the FpHXK1 serves as a glucose sensor depending on its catalytic activity.

### 3.6 Expression analysis of the *FpHXK1* under normal or drought stress conditions

The cis-acting regulatory element in the promoter region of the *FpHXK1* gene was investigated (**Supplementary Table 4**). A total of 181 cis-acting elements were found, including those associated with light responsiveness (AE-box, GT-1 motif, G-box), abscisic acid responsiveness (ABRE), drought-inducibility (ABS), anaerobic induction (ARE), meristem expression (CAT-box), and the binding of AT-rich DNA binding protein (ATBP-1). The most diverse ones are those related to the light-responsiveness. Additionally, we identified an element associated with the expression of meristems, which are closely related to root and stem growth and thickening. Notably, the *FpHXK1* promoter contains two stress-responsive and one ABA-responsiveness element. We speculated that *FpHXK1* might play an important role in strawberry plant’s resistance to abiotic stresses, like drought and anaerobic stress.

**Figure 6:**
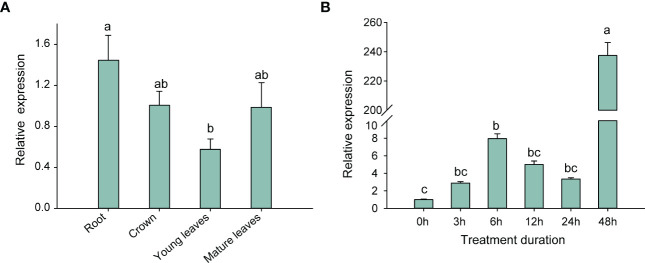
Expression levels of strawberry *HXK1* in different tissues and variation under 20% PEG-6000 simulated stress condition. **(A)** Relative levels of *FpHXK1* transcripts in different tissues based on RT-qPCR. **(B)** Relative *FpHXK1* gene expression level in response to 20% PEG-6000 exposure. Values were presented as mean ± SEM of at least three biological repetitions. Statistically significant differences among samples were tested by Tukey’s test, significantly different (p< 0.05) were indicated by different letters, as determined by a two-way analysis of variance.

To validate the assumption, we analyzed the relative expression of the *FpHXK1* gene in the roots, crowns and leaves of strawberry plants under normal growth conditions (**Figure 6A**), and investigated the changing trend of *FpHXK1* in the whole strawberry plantlets under the PEG-simulated drought conditions (**Figure 6B**). The relative gene expression of the *FpHXK1* was highest in the roots, followed by crowns and mature leaves. Under drought conditions, the expression level of *FpHXK1* firstly increased and then decreased in the first 24 hours. However, a burst of increase was observed 48 h after treatment, when the wilting of leaves began to occur. We speculated that under short-term mild drought and osmotic stress, FpHXK1 was involved throughout the recovery process.

### 3.7 The role of the hexokinase activity in drought tolerance

**Figure 7:**
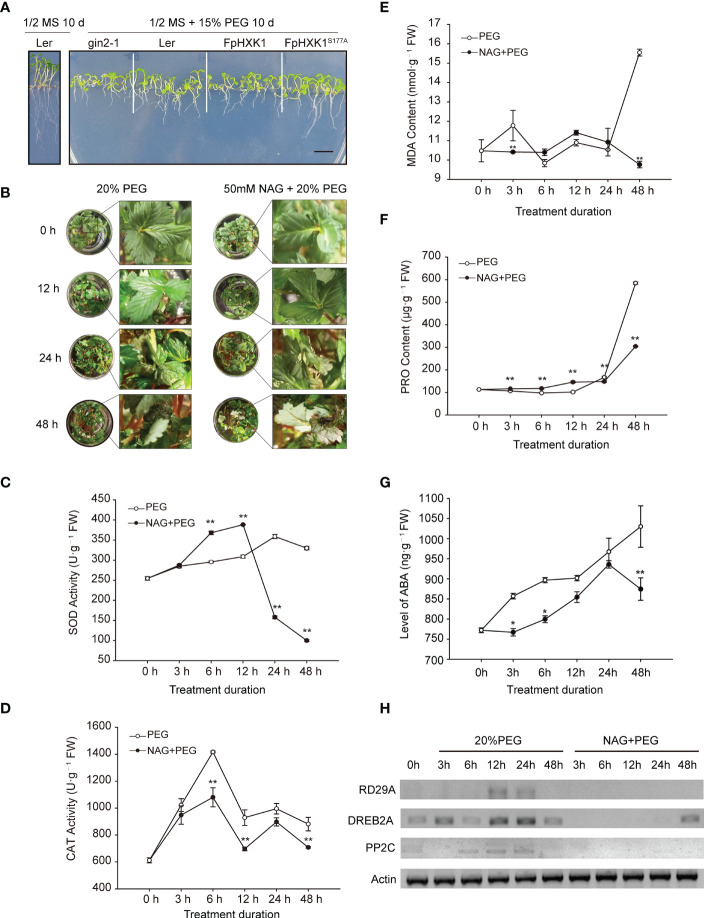
The role of strawberry *FpHXK1* on drought stress responses in *A. thaliana* and strawberry plants. **(A)** WT (*Ler*) and transgenic seedlings germinated on MS medium with 15% PEG-6000 for 10 days. Scale bar, 0.5 cm; **(B)** Plant phenotype of the strawberry plantlets exposed to 20% PEG-6000 or combination of 50 mM NAG and 20% PEG-6000 for 0, 12, 24, 48 h respectively. **(C–G)** Altered levels of SOD, CAT, MDA, PRO and ABA in strawberry plants. Vertical bars represent SEM. Asterisks indicate significant differences between plants in which the HXK activity was inhibited by NAG and in the control plants (*p< 0.05; **p< 0.01). **(H)** Transcript levels of drought-induce genes (*RD29A*, *DREB2A* and *PP2C*), *FpActin* was used as a reference.

To further determine the role of FpHXK1 in response to drought stress, the transgenic plants, *gin2-1*, and WT-type (*Ler*) Arabidopsis seedlings were treated with 15% PEG-6000. After 10 days of treatment, a strong reduction of growth was observed in all genotypes upon the osmotic stress when compared with wild-type under normal conditions. The *gin2-1* suffered the most, with severe root and cotyledon inhibition. The growth status of the WT and *35S::FpHXK1^S177A^* seedlings was similar. Both were relatively better than that of the *35S::FpHXK1* plants, regarding the root and hypocotyl growth (**Figure 7A**).

The WT strawberry plants were treated with 20% PEG-6000, in the presence or absence of N-acetylglucosamine (NAG), a known hexokinase inhibitor which was commonly used to investigate the role of plant hexokinases (Han et al., 2022; Li et al., 2022). After 12 hours, the plants began to show obvious wilting. After 24 hours, irreversible wilting and leaf necrosis began to appear in both treatments. These phenomena were more evident at 48 h (**Figure 7B**). From the phenotype of plants, the inhibition of hexokinase aggravated this deterioration. We analyzed the activity of the superoxide dismutase (SOD) and catalase (CAT), the content of malondialdehyde (MDA), and proline (Pro) in the plantlets at 0 h, 3 h, 6 h, 12 h, 24 h, and 48 h after treatment. The activity of the SOD in the PEG treated plants showed a gradual increase. The activity of SOD in the NAG-inhibited plants was significantly higher during the early stages (before 12 h), but a sharp decline occurred at the later stages (**Figure 7C**). The activity of the CAT in both treated plants showed fluctuations, but it was lower in the NAG-inhibited plant throughout the experiment (**Figure 7D**). The accumulation of MDA in the kinase- active or inhibited plants was relatively stable in the first 24 h (**Figure 7E**). However, a sharp increase of MDA was observed in the PEG treated plants, indicating of injuries of the plasma membrane, a phenomenon that was not observed in the kinase-inhibited plants. Proline (Pro) accumulation is a common physiological response for plants in response to drought stress (Singh et al., 1972). A lower concentration of Pro was accumulated in the PEG treated plants than in those NAG-PEG treated ones during the first 12-hour stress. This trend was reversed due to a more pronounced rise of Pro in the kinase-active plants (**Figure 7F**). The content of stress-related phytohormone ABA was determined too. ABA starts to accumulate three hours after drought treatment in the plants, and was still increasing at the end of the experiment (48 h). However, when treated with NAG, this response was significantly repressed (**Figure 7G**). We also tested the changes of the transcripts of drought-induced genes (RD29A, DREB2A, PP2C) (Li et al., 2021) using semi-quantitative RT-PCR (**Figure 7H**). The transcriptions of these genes were all repressed when the kinase activity of HXK was inhibited, in contrasting to the observed induction in the PEG treated plants. These results showed that the inhibition on the HXK kinase activity weakened the plant’s responses to drought stress.

### 3.8 Transient overexpression of *FpHXK1*/*FpHXK1^S177A^* impacts the strawberry fruit’s sugar metabolism

**Figure 8:**
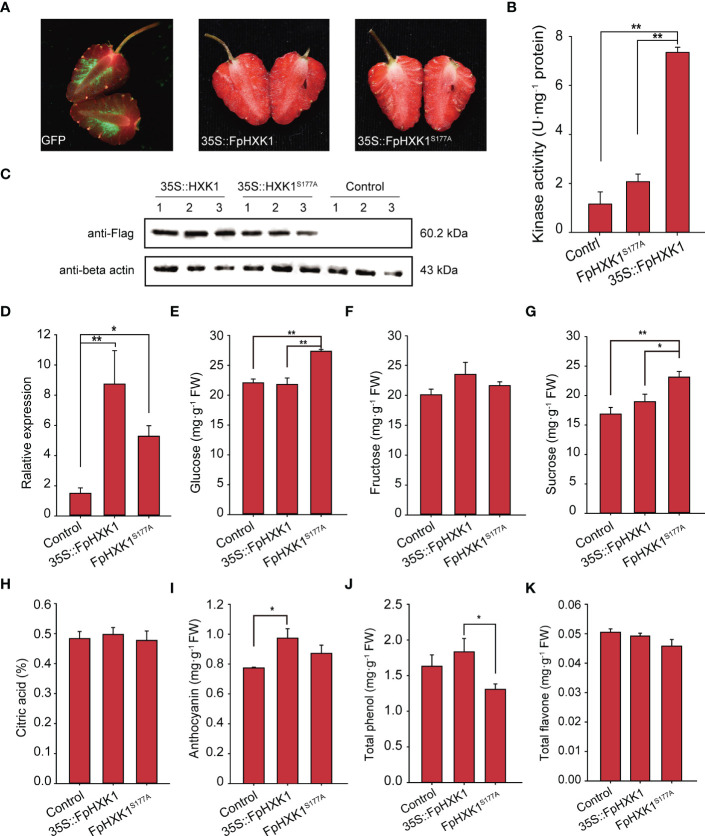
Effects of transient overexpression of the *FpHXK1* and *FpHXK1^S177A^* on the strawberry fruit primary and secondary metabolites. **(A)** Phenotype of the fruits infiltrated with *mGFP* empty vector, *35S::FpHXK1*, and *35S::FpHXK1^S177A^* constructs. The arrows point to the injection sites. **(B)** Kinase activity assay of the extracts of recombinant FpHXK1 and FpHXK1^S177A^. ** indicates significant difference at the level of *p*< 0.01. **(C)** Western blot analysis of the recombinant protein of FpHXK1 and FpHXK1^S177A^ in strawberry fruits. **(D)** Relative gene expression of the *FpHXK1* and *FpHXK1^S177A^* seven days after infiltration. The *FaActin* gene was used as a reference. Values were expressed as the mean ± SEM of 30 fruits of in each group. **(E–K)** The changes of the primary and secondary metabolites in strawberry fruits due to the OE of *FpHXK1* or the mutant. Statistically significant differences among samples were tested by Tukey’s test (**p< 0.01, *p< 0.05).

Since the signaling of FpHXK1 was linked to its kinase activity, which was directly involved in the sugar metabolism in the glycolysis pathway, it is interesting to check what impact could FpHXK1 and its kinase impaired mutant bring to the strawberry fruits. We first investigated the expression level of FpHXK1 in the fruits using our previously sequenced transcriptome data (Bai et al., 2019). Among the four identified FpHXKs, the highest transcription level was observed for FpHXK1 in the fruits (**Supplementary Figure 6**). Then we transiently over-expressed the gene in strawberry fruits and determined the major sugar content and four common nutrients of the fruits. After seven days of infection, the control GFP signals were evident in the flesh tissue, indicating that the constructs were successfully infiltrated and expressed (**Figure 8A**). Using the extracted raw protein of the fruit sample, both the hexokinase activity test (**Figure 8B**) and western blot assay results (**Figure 8C**) confirmed the successful production of the infusion FpHXK1-Flag protein. Gene expression analysis showed that the transcript abundances were four to eleven-fold higher than that of the control plants (**Figure 8D**). The infiltrated fruits were phenotypically similar between the two constructs. Phytochemical test demonstrated that overexpression of the mutant *FpHXK1^S177A^* significantly promoted the accumulation of glucose and sucrose in the fruit (**Figures 8E, G**). The glucose content increased by 24% and 26% relative to the mock control and *35S::FpHXK1* fruits respectively. The sucrose content was also significantly higher (**Figure 8G**). The anthocyanin content was promoted by both *35S::HXK1* and *35S::HXK1^S177A^*, more pronounced in the former (**Figure 8I**). In contrast, the total phenol content was significantly lower in the catalytically inactive *FpHXK1-*OE fruits (**Figure 8J**). Overexpression of both genes did not significantly impact the accumulation of fructose, citric acid, and the total flavone in our test (**Figures 8F, H, K**).

## 4 Discussion

### 4.1 The gene expansion of *HXKs* in green plants

Hexokinase is the gate-way enzyme of the glycolytic pathway which has been identified in an array of species in all living organisms. Based on the phylogenetic analyses of a limited number of plant’s HXKs, Olsson et al. (2003) classified the gene family members into two large groups, namely type A, which have plastid signal peptides in the N-terminal, and type B, which possess membrane anchors instead. Later, through the investigation of the whole genome sequence of the moss *P. patens*, two novel types named type C and type D HXKs were defined (Nilsson et al., 2011). In this study, we expanded the scope of the species included and again suggested the concerted evolution of the hexokinase gene family within the *P. patens* but not in other species (**Figure 2**). However, three phylogenetic clades (S1 to S3) were obtained instead of two. The unicellular green algae (*K. nitens*) at the basal position of the tree has only one member of hexokinase in the genome, as reported by Ulfstedt et al. (2018), implying that from one common ancestor gene that all green plants’ HXKs derive. Different gene expansion/contraction events must have occurred in different plant lineage. In line with the previous findings, we have identified 10 HXKs in rice (Cho et al., 2006a), 10 HXKs in pear (Yu et al., 2017), and 9 in apple (Zhu et al., 2021), 6 HXKs in *A. thaliana* (Karve et al., 2008), 4 in *F. vesca* and *F. pentaphylla* (**Supplementary Table 1**). The gene number differences were mainly attributed to the gene divergence in the S3 clade.

AtHKL3, which is far diverged from the other *Arabidopsis* HXK members, was assigned into one of the subclades in the S3 group. It was proposed that the members of this clade were diverged from other members prior to the differentiation of dicots and monocots (Karve, 2008). However, we argued this assumption because no monocots HXK members represented by *O. sativa* in our study were assigned into this group (AtHKL3). Instead, all seven rice HXKs in the S3 clade concerted, forming a unique subclade (**Figure 2**). There might be an independent gene duplication in the dicots from which the other HXKs like the HXK1 and HXK2 originated, as proposed by Cho et al. (2006a). Moreover, there are no strawberry HXK members belonging to the S2 subclade, in which AtHKL1 and AtHKL2 reside. AtHKL1 is a negative regulator of plant growth. Its function depends on the presence of AtHXK1 (Karve, 2008). Both AtHKL1 and AtHKL2 lack kinase activity and cannot phosphorylate hexoses. In our study, the signaling route of FpHXK1/FvHXK1 was dramatically distinct from that of AtHXK1, which might lead to the loss of such HKLs in strawberry.

### 4.2 FpHXK1 acts as a glucose sensor depending on its kinase activity

It has long been pointed out that at least three distinct glucose signaling transduction pathways exist based on the revealed roles of plant HXK1 (Xiao et al., 2000). The catalytically inactive AtHXK1 (G104D and S177A) in a loss-of-function mutant *gin2-1* could still mediate the growth repression response to high glucose (6%), for the first time demonstrated the possibility of uncoupling the signaling function of HXK1 from its catalytic activity (Moore et al., 2003). Similar results were later observed in rice hexokinases OsHXK5 and OsHXK6 (Cho et al., 2009). Through the alternations of the downstream sugar signaling pathway genes, mainly the photosynthesis genes like *CAA* or *CAB* genes, the OsHXK7 but not the catalytically inactive alleles could complement the *gin2-1* mutant (Kim et al., 2016). Likewise, the restoration of the glucose signaling of the unique HXK in the charophyte alga relies on its sugar phosphorating activity (Ulfstedt et al., 2018). In our study, the *35S::FpHXK1* allele recovered the glucose repression phenotype in *gin2-1* plants, whereas the catalytically impaired S177A allele did not, which even promoted the root growth (**Supplementary Figure 4B**). The inhibition of the *CAB* gene was abolished through complementation with *35S::FpHXK1^S177A^* but maintained in those with *35S::FpHXK1*. These results support the notion that FpHXK1 functions in sugar sensing and signaling *via* a kinase-dependent pathway, which is distinct from the sugar sensor AtHXK1.

### 4.3 The role of FpHXK1 in drought tolerance

**Figure 9:**
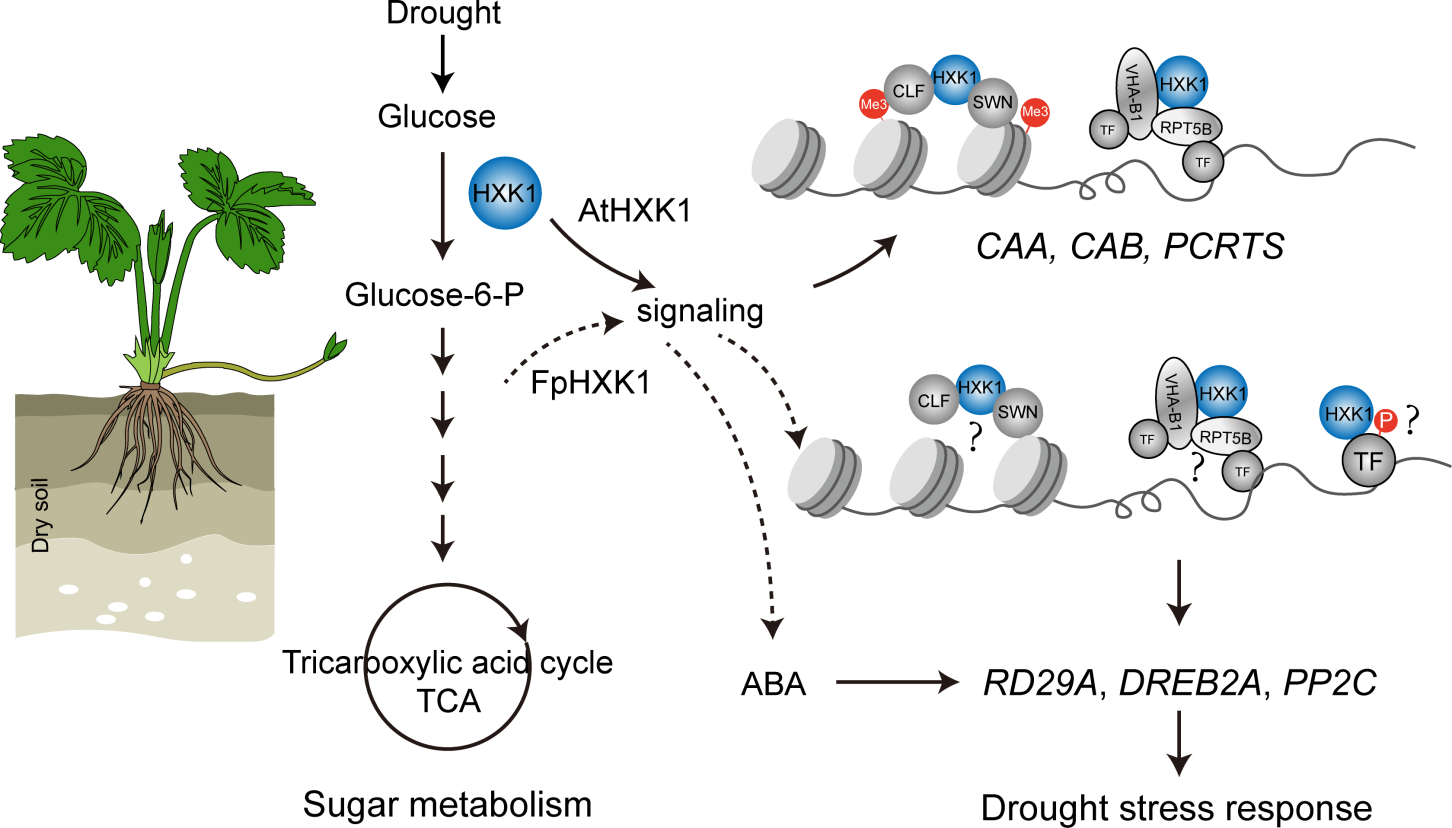
A schematic diagram showing the mechanism through which the FpHXK1 participates in drought stress response and sugar metabolism in strawberry plants. Me3: histone H3 lysine27 trimethylation (H3K27me3); CAA, carbonic anhydrase; CAB, chlorophyll a/b binding protein; PCRTS, CLF, Curly Leaf; SWN, Swinger; VHA-B1, Vacuolar proton pump subunit; RPT5B, 19S regulatory particle of proteasome subunit; TF, Transcription factors; DREB2A, Dehydration-responsive element binding protein2a; PP2C, Protein phosphatases type 2C.

Unfavorable environmental conditions like drought have a negative impact upon the plant photosynthesis assimilation process, reprogramming the sugar synthesis and distribution (Kaur et al., 2021). Several records have revealed the involvement of HXK, the first catalytic enzyme of the sugar metabolism in plant stress responses. MdHXK1 can phosphorylate the Na^+^/H^+^ exchanger MdNHX1 to enhance the tolerance to salt stress (Sun et al., 2018). While NbHXK1 in tobacco, which governs the cell programmed death in the tobacco leaves, promotes plants’ resistance to the methyl viologen-induced oxidative stress and pathogen infection (Sarowa et al., 2008). Inhibition of HXK by NAG decreased the phenylpropanoid metabolic pathway and resistance to Brown Rot has been documented in peach fruits (Han et al., 2022). Through the NAG inhibition experiments, it is evident that the antioxidant enzyme system, stress-induced ABA accumulation, and the selected typical drought responsive genes were all directly affected (**Figure 7**), indicating that the hexokinase activity of HXKs were indispensable for strawberry’s stress response (summarized in **Figure 9**). For the FpHXK1, its signaling function also relies on its hexokinase activity. We still do not know whether and how sugar signals participate in these reactions. Deep investigation into the underlying molecular mechanism will be helpful in exploring new strategies to solve such abiotic stresses.

### 4.4 FpHXK1 impacts sugar and secondary metabolism in strawberry fruits

In strawberry cultivars with high-sugar content, sucrose is the main sugar component, while fructose dominates in cultivars with low-sugar content. Both sugars are substrates of plant hexokinases. In 2018, Lee et al. (2018) found that the *FaHXK1* was highly expressed in strawberry cultivars with high-sugar content. We still do not know whether it was related to the sugar metabolism or signaling pathway. Several sugar-metabolism-related genes, exemplified by the *MdFRK2* (Su et al., 2021) and *phosphoenolpyruvate carboxykinase* (*PEPCK*) (Osorio et al., 2013), were reported to be directly linked to the sugar accumulation of plants. When we transiently overexpressed the catalytically activate *FpHXK1* in strawberry fruits, the glucose, and sucrose contents were not changed compared with the empty vector control, indicating robust sponge effects of the sugar metabolites of the glycolysis pathway in the fruits at the tested stage. Conversely, the elevation of the signaling ineffective allele *FpHXK1^S177A^* brought a more pronounce effect to the sugars (**Figures 8E, G**), possibly through a competitive way to the canonical HXK1 among other unknown mechanisms. The catalytic-activity-independent signaling of the AtHXK1 is partially through the interaction with VHA-B and RPT5B (Cho et al., 2006b), or *via* CLF and SWN (Liu et al., 2022). Such signaling circuit may not be applicable to the catalytic-activity-dependent HXKs, FpHXK1 in this study for example, since both the WT AtHXK1 and AtHXK1^S177A^ could interact with CLF and SWN (Liu et al., 2022). Noteworthy, MdHXK1 as well as the mutant allele could phosphorylate and stabilize bHLH3 to promote anthocyanin biosynthesis (Hu et al., 2016). Transiently overexpression of the *FpHXK1* in strawberry fruits also elevated the anthocyanin content (**Figure 8I**) in the current study. Similar regulation mechanism might exist here. However, whether it holds true for the sugar content modulation still needs further validation.

## 5 Conclusion

In this study, four HXKs were firstly identified in the genome of *F. vesca* and *F. pentaphylla*. *FpHXK1* (*Fpe07G005790.1*), which shared the highest identity with *AtHXK1* was able to restore the glucose sensitivity and developmental defects of the Arabidopsis *gin2-1* mutant, but not its kinase activity-deficient mutant (*FpHXK1^S177A^*). The inhibition on the HXK kinase activity weakened the strawberry plant’s responses to the PEG-simulated drought stress. Transient overexpression of *FpHXK1* and its kinase-impaired mutant differentially affected the level of glucose, sucrose, anthocyanins, and total phenols in strawberry fruits. All these results indicated that the FpHXK1, acting as a glucose sensor, was involved in drought stress response and sugar metabolism depending on its kinase activity.

## Data availability statement

The raw data supporting the conclusions of this article will be made available by the authors, without undue reservation.

## Author contributions

Conceptualization, QC and HT; Methodology, RW, XL, JH, and LP; Resources, YW, YL, and XW; Software, RW, YXL, YTZ, and YZ; Data curation and visualization, RW, ML, AM, and WH; Writing-original draft preparation, RW; Project administration, QC; Funding acquisition, QC and HT. All authors contributed to the article and approved the submitted version.
