# Supplementary material for: Hexokinase1: A glucose sensor involved in drought stress response and sugar metabolism depending on its kinase activity in strawberry
Source: Front Plant Sci. 2023 Jan 27;14:1069830. doi: 10.3389/fpls.2023.1069830 (PMC9911861; doi:10.3389/fpls.2023.1069830)
Supplement: Supplementary file 1 [file DataSheet_1.docx]

Supplementary Material

**Hexokinase1: A Glucose Sensor Involved in Drought Stress Response and Sugar Metabolism Depending on Its Kinase Activity in Strawberry**

**Runqin Wu^1^, Ximeng Lin^1^, Jinwei He^1^, Ailing Min^1^, Li Pang^1^, Yan Wang^2^, Yuanxiu Lin^2^, Yunting Zhang^2^, Wen He^2^, Mengyao Li^1^, Yong Zhang^1^, Ya Luo^1^, Xiaorong Wang^2^, Haoru Tang^2,*^, Qing Chen^1,*^**

^1^ College of Horticulture, Sichuan Agricultural University, Chengdu 611130, Sichuan, China

^2^ Institute of Pomology and Olericulture, Sichuan Agricultural University, Chengdu 611130, Sichuan, China

**Supplementary Table 1** Primers used in this study

| Targeted gene | Primer sequence | Note |
| --- | --- | --- |
| FpHXK1 | Fwd: 5’-CTTTGTAGTCGACGAGCTCGACTGAATCCTCTACCCCAAGGT-3’  Rev: 5’-GACGATGACAAGCTTGGATCCATGGGGAAGGTGGCG-3’ | Gene cloning |
| FpHXK1^S177A^ | Fwd：5'-ACACGGGAAATGCGAAGGTAAAACCCCAGCTCC-3'  Rev: 5'-GGGTTTTACCTTCGCATTTCCCGTGTGGC-3' | SOE-PCR |
| FpHXK1 | Fwd: 5‘-TTGTATCATTGACCAAAGCTGTAACACG-3‘  Rev: 5’-GTATTGAAGATGCGGTTGAGCAAGATG-3’ | RT-qPCR |
| FaActin | Fwd: 5’-GCTAATCGTGAGAAGATGAC-3’  Rev: 5’- AGCACAATACCAGTAGTACG-3’ | RT-qPCR |
| AtTUB3 | Fwd: 5’-TTCACAGCAAGCTTACGGAGGTCA-3’  Rev: 5’-TGGTGGAGCCTTACAACGCTACTT-3’ | RT-qPCR |
| AtCAA | Fwd: 5'-TGAATACGCTGTCTTGCACC-3'  Rev: 5'-TGTGATGGTGGTGGTAGCGA-3' | RT-PCR |
| AtCAB | Fwd: 5'-ATGGCCACTTCAGCAATCCAA-3'  Rev: 5'-CACAACTTGACACGCCCATAT-3' | RT-PCR |
| AtUBQ10 | Fwd: 5'-GATCTTTGCCGGAAAACAATTGGAGGATGGT-3'  Rev: 5'-CGACTTGTCATTAGAAAGAAAGAGATAACAGG-3' | RT-PCR |
| AtHXK1 | LP: 5’-GATTTCTACTGGTGCTGCTGG-3’  RP: 5’-CTCTGCTGCTGGAATCTATGG-3’  LB_6313R: 5’-TCAAACAGGATTTTCGCCTGCT-3’ | *gin2-1* phenotyping |
| HygR | Fwd: 5'-CAGGGTGTCACGTTGCAAGAC-3'  Rev: 5'-TCCATACAAGCCAACCACGGC-3' | PCR screening |
| FpHXK1 | Fwd: 5’-GACCAAAGCTGTAACACGCATATCC-3’  Rev: 5’-AGAAGTGTGACACGCCGATTG-3’ | PCR screening |
| FpRD29A | Fwd: 5’-GATGTCACTGGTGCTGGGAAGT-3’  Rev: 5’-CCTTTAAACTTGGACATCACAGCGG-3’ | RT-PCR |
| FpDREB2A | Fwd: 5’-AGATTCGAGAGCCGAACAGAGGTA-3’  Rev: 5’-GTATGAGCGTGATCCATAGGAACCT-3’ | RT-PCR |
| FpPP2C | Fwd: 5’-GCTGTTCATCATCAAGGGTGGCA-3’  Rev: 5’-CAAACCATCACTCGCCAGTATCAGA-3’ | RT-PCR |
| FpActin | Fwd: 5’-AATGAGCTTCGTGTTGCTCCTGA-3’  Rev: 5’-GGAGCTGCTCTTGGAGGTCT-3’ | RT-PCR |

**Supplementary Table 2** Information of HXK genes identified in 19 species

| Species | HXK gene ID (protein coding) |
| --- | --- |
| *Arabidopsis thaliana* | AT4G29130.1(AtHXK1), AT2G19860.1(AtHXK2), AT1G47840.1(AtHXK3), AT1G50460.1(AtHKL1), AT3G20040.1(AtHKL2), AT4G37840.1(AtHKL3) |
| *Fragaria vesca* | FvH4_1g06270.t1(FvHXK1),FvH4_6g32470.t2(FvHXK2), FvH4_2g38530.t1(FvHXK3), FvH4_1g18730.t1(FvHXK4) |
| *Fragaria pentaphylla* | Fpe07G005790.1(FpHXK1), Fpe01G031800.1(FpHXK2), Fpe04G039540.1(FpHXK3), Fpe07G018400.1(FpHXK4) |
| *Malus Domestica* | MD15G1197000(MdHXK1), MD17G1183100, MD09G1202200, MD02G1066500, MD11G1188100, MD15G1012900, MD03G1170700, MD15G1305100, MD02G1194700 |
| *Pyrus × bretschneideri* | XM_009354280.1(PbHXK1), XM_009340459.1, XM_009367798.1, XM_009377080.1, XM_009338754.1, XM_009345525.1, XM_009336810.1, XM_009356359.1, XM_009356362.1, XM_009377604.1 |
| *Prunus persica* | Prupe.7G218800.1(PrpHXK1), Prupe.3G057800.1(PrpHXK2), Prupe.4G256200.1, Prupe.1G366000.1, Prupe.6G212100.1 |
| *Vitis vinifera* | VIT_211s0016g03070.1(VvHXK1), VIT_209s0002g03390.1, VIT_206s0061g00040.2, VIT_218s0001g14230.1, VIT_200s0824g00010.1 |
| *Solanum Lycopersicum* | Solyc03g121070.3.1(SlHXK1), Solyc06g066440.3.1, Solyc12g008510.2.1, Solyc11g065220.2.1, Solyc04g081400.3.1, Solyc02g091830.3.1 |
| *Solanum tuberosum* | Soltu.DM.06G023000.1(StHXK1), Soltu.DM.03G035820.1(StHXK2), Soltu.DM.11G019290.1, Soltu.DM.04G036520.2, Soltu.DM.12G025470.2, Soltu.DM.02G027090.1 |
| *Spirodela polyrhiza* | Spipo2G0121500, Spipo6G0009800, Spipo16G0021200 |
| *Oryza sativa* | Os05g44760.1(OsHXK5), Os01g53930.1, Os05g45590.1, Os05g09500.1(OsHXK7), Os01g52450.1, Os07g09890.1, Os07g26540.1, Os01g71320.1, Os01g09460.1, Os05g31110.1 |
| *Chlamydomonas reinhardtii* | Cre02.g117500.t1.2 |
| *Physcomitrium patens* | Pp3c14_6150V3.1(PpHXK1), Pp3c19_20120V3.1(PpHXK2), Pp3c22_9450V3.1, Pp3c21_19280V3.1, Pp3c18_12510V3.1, Pp3c2_11350V3.1, Pp3c10_8650V3.1, Pp3c8_18980V3.1, Pp3c1_5000V3.1 |
| *Nicotiana benthamiana* | NbD046380.1(NbHXK1), NbD012210.1, NbD036894.1, NbD044815.1, NbD000314.1, NbD010989.1, NbD018170.1 |
| *Manihot esculenta* | 16G109200.2(MeHXK2), Manes.04G134700.1.p, Manes.14G005400.1.p, Manes.06G161400.1.p, Manes.18G014000.2.p, Manes.03G026700.1.p, Manes.12G119601.2.p |
| HXK3s of *Prunus* | HXK3MazzardF12/1, HXK3Mariana2624 |
| *Saccharomyces cerevisiae* | YGL253W, YFR053C |
| *Caenorhabditis elegans* | chrIV_pilon.g11771, chrI_pilon.g2247, chrIV_pilon.g9685 |
| *Homo sapiens* | P19367(HK1), P52789(HK2), P52790(HK3), P35557(HK4) |

**Supplementary Table 3** The information of motifs in *F. vesca*, *F. pentaphyalla*, and *A. thaliana*

| Motif | Pfam domain | sequence | Description |
| --- | --- | --- | --- |
| Motif1 | DUF5957 | VTAAVACAAAAVLVRRRMKSK | PF19382, family of unknown function |
| Motif2 | Hexokinase_1 | WARVTAILKEFEEDCATPIPKLRQVADAMTVEMHAGLASEG | PF00349, hexokinase |
| Motif3 | Hexokinase_1 | KLKMJISYVDNLPTGBEKGLFYALDLGGTNFRVLRVQLGGK | PF00349, hexokinase |
| Motif4 | Hexokinase_1 | ERVIKQEFEEVSIPPHLMTGT | PF00349, hexokinase |
| Motif5 | Hexokinase_1 | SEELFDFIASELAKF | PF00349, hexokinase |
| Motif6 | Hexokinase_1 | EGEEFHLPPGRKRELGFTFSFPVKQTSISSGTLIKWTKGFS | PF00349, hexokinase |
| Motif7 | Hexokinase_1 | DDTVGKDVVAELNKALEKHGL | PF00349, hexokinase |
| Motif8 | Hexokinase_1 | DMRVSALVNDTVGTLAGGRYYBPDVVAAVILGTGTNAAYVERADAIPKWH | PF00349, hexokinase |
| Motif8 | Hexokinase_2 | DMRVSALVNDTVGTLAGGRYYBPDVVAAVILGTGTNAAYVERADAIPKWH | PF03727, hexokinase |
| Motif9 | Hexokinase_2 | PKSGEMVINMEWGNF | PF03727, hexokinase |
| Motif10 | Hexokinase_2 | SSHLPLTEYDHSLDAESLNPGEQIFEKIISGMYLGEIVRRVLLKM | PF03727, hexokinase |
| Motif11 | Hexokinase_2 | GDTVPPKLSTPFILRTPDMSAMHQDTSPDLKVVGSKLKBIL | PF03727, hexokinase |
| Motif12 | Hexokinase_2 | ISESSLKVRKVVVEVCDIVATRGARLAAAGILGILKKLGRDTK | PF03727, hexokinase |
| Motif13 | Hexokinase_2 | QKKTVVAM | PF03727, hexokinase |
| Motif14 | Hexokinase_2 | DGGLYEHYTEFREYMESALRELLGDEVAEHVVIEHSNDGSGIGAALLAAS | PF03727, hexokinase |
| Motif15 | Trypan_PARP | QMKDIDQSQMQDSDPDVEAKPEPEPEPEF | PF05887, procyclic acidic repetitive protein (PARP) |

**Supplementary Table 4** The cis-elements in the promoter regions of the FvHXK1 gene

| Name | Location | sequence | function |
| --- | --- | --- | --- |
| MBS | +15 | CAACTG | MYB binding site involved in drought-inducibility |
| ARE | -1224, +1833 | AAACCA | anaerobic induction element |
| CAT-box | +1333 | GCCACT | meristem expression |
| ABRE | -1770, -1772, -1837,  -1970 | TACGTGTC  ACGTG | abscisic acid responsiveness |
| AE-box | -565, +1267 | AGAAACAA  AGAAACTT | light responsive element |
| GT1-motif | +552, -697, -1802 | GGTTAA | light responsive element |
| G-box | -1772 | TACGTG | light responsiveness |
| ATBP-1 | -1232 | ATAGAAATCAA | the binding site of AT-rich DNA binding protein |


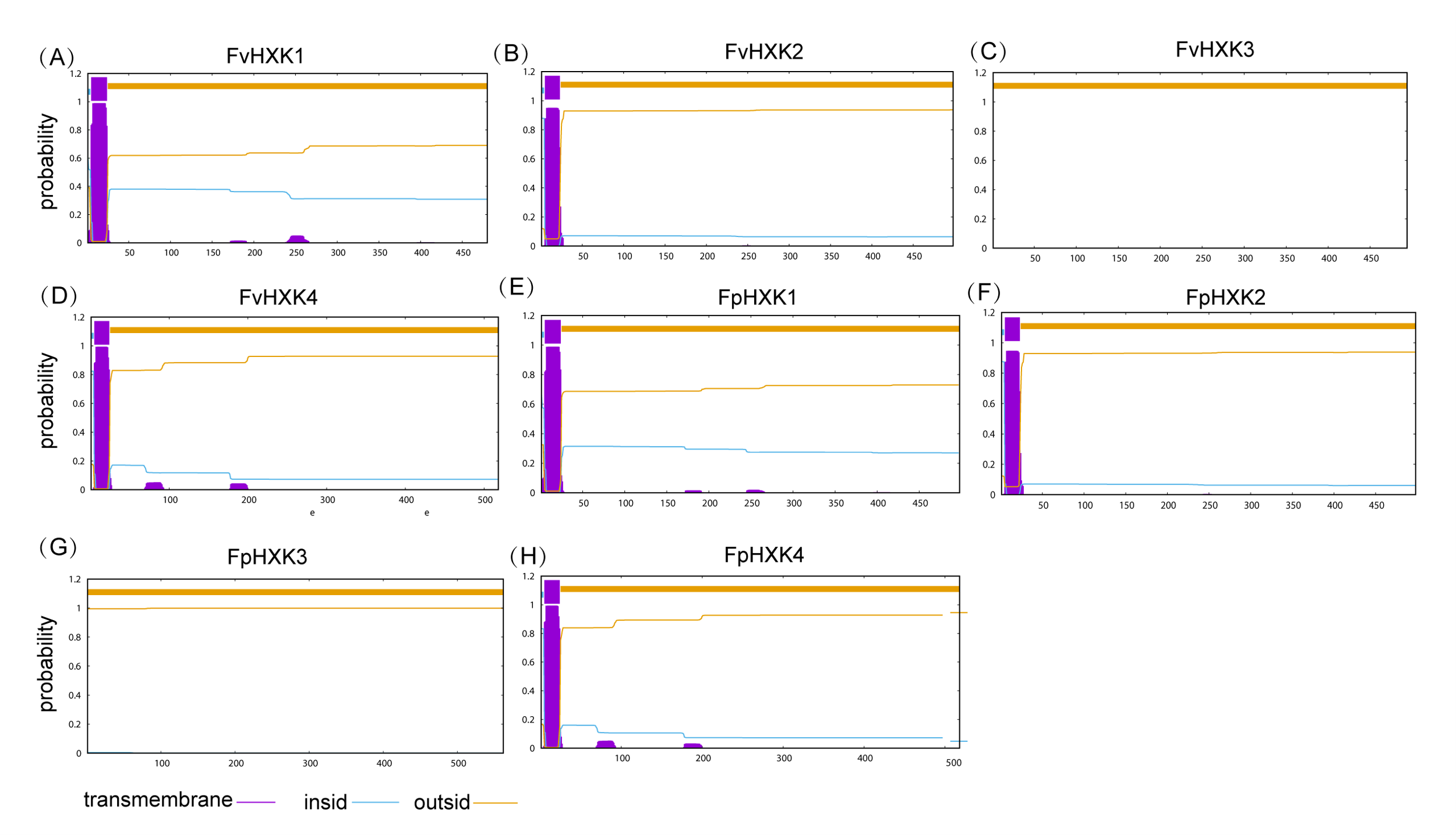


**Supplementary Figure 1** TMHMM posterior probabilities for FvHXKs and FpHXKs. The image was generated using TMHMM-2.0.

**
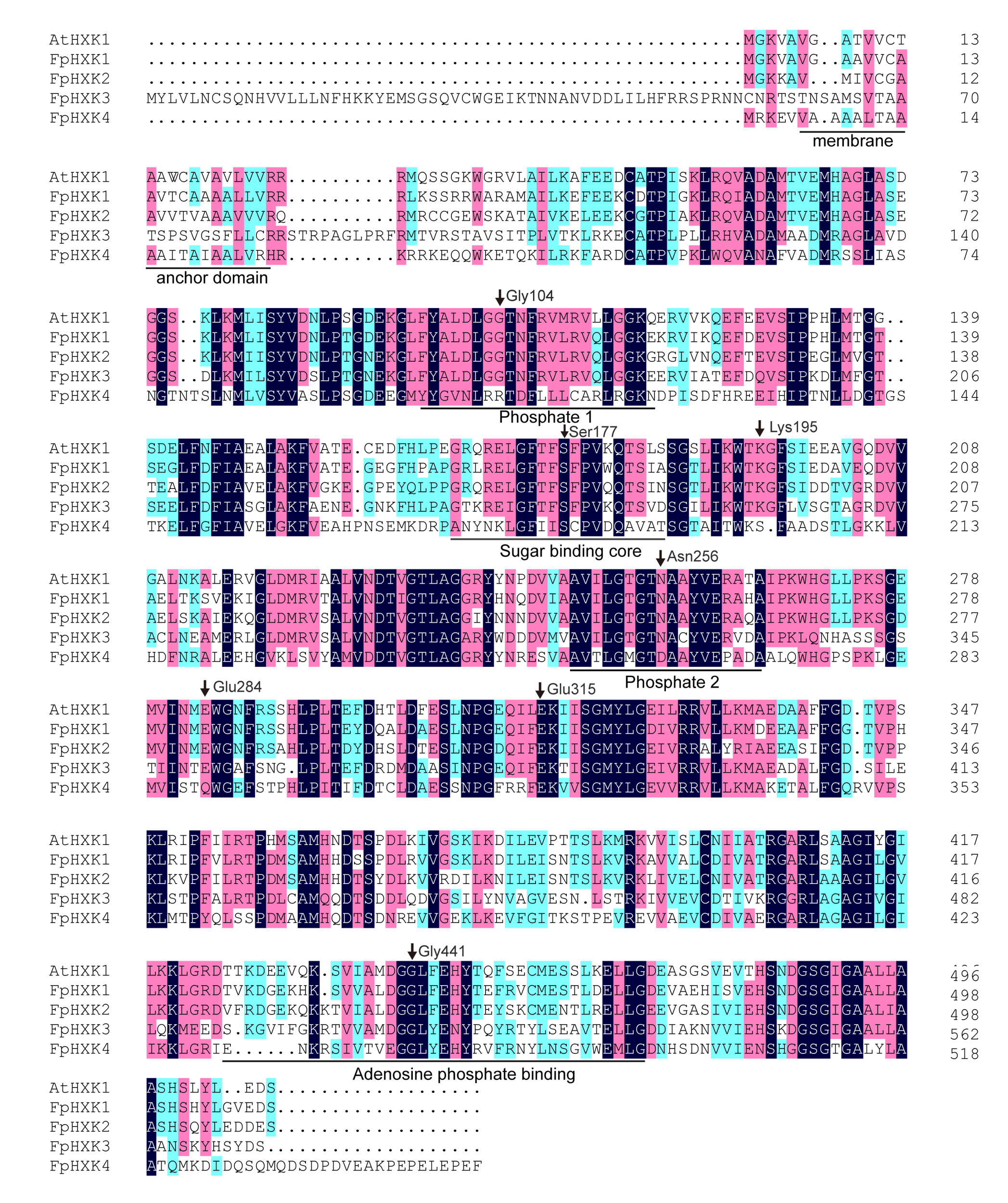
**

**Supplementary Figure 2** Amino acid alignment analysis of the identified strawberry (*Fragaria pentaphylla*) HXKs with AtHXK1.

**
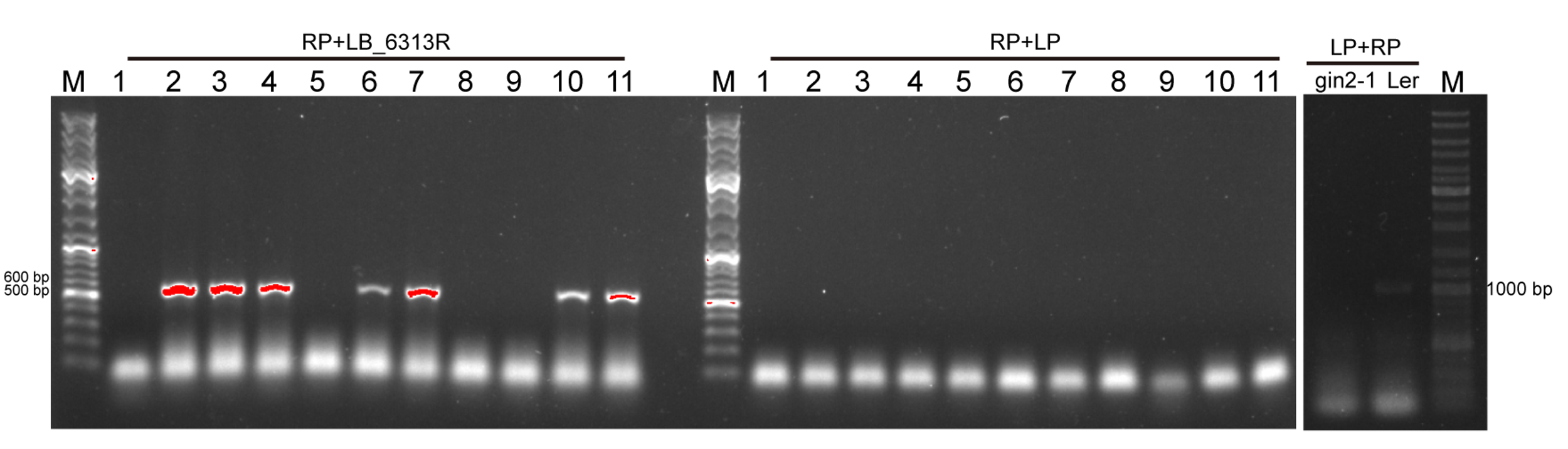
**

**Supplementary Figure 3** PCR screening of the *Arabidopsis thaliana* *gin2-1* plants.

**
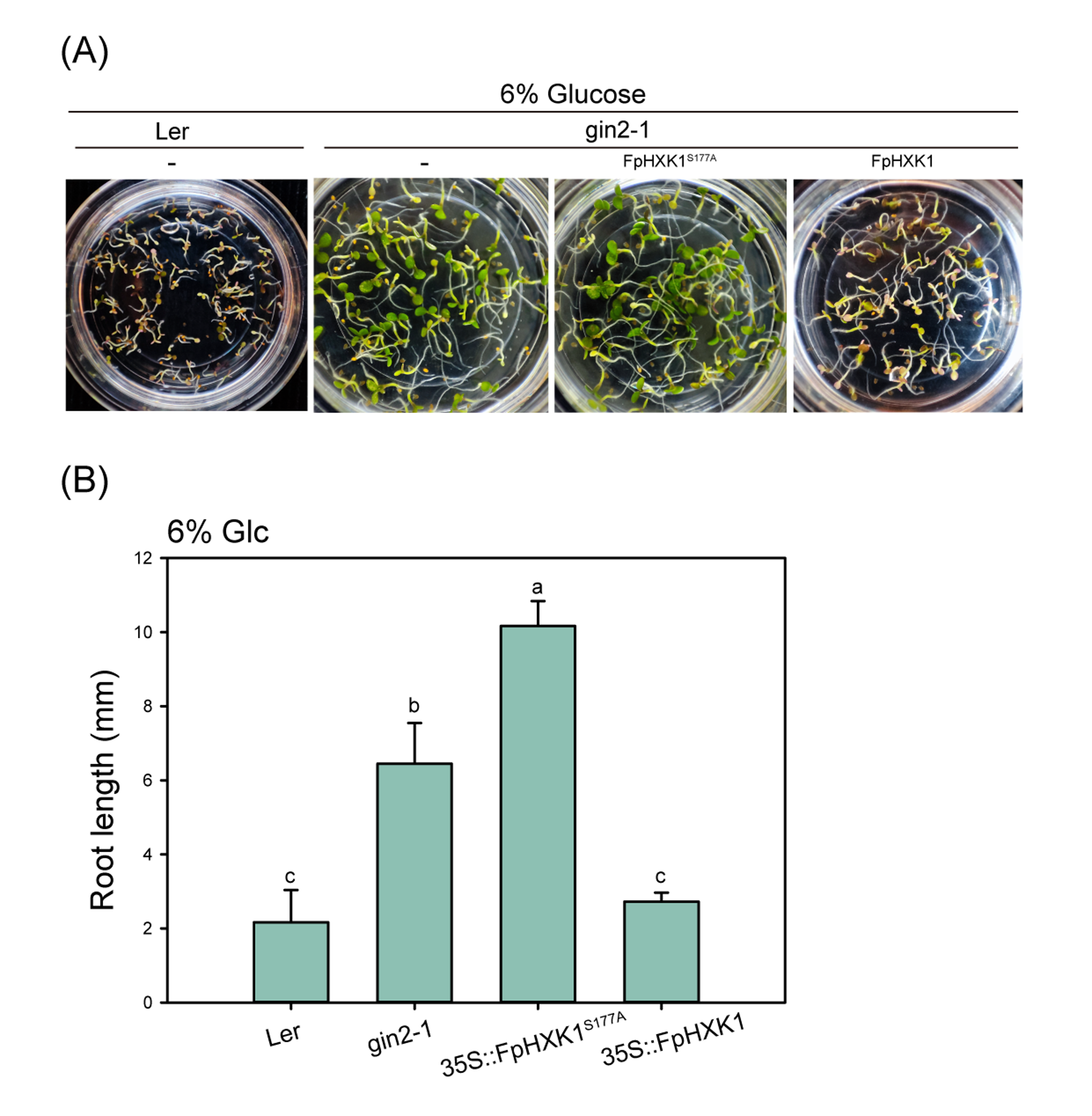
**

**Supplementary Figure 4** (A) Seedling-growth of transgenic, gin2-1, and wild-type Arabidopsis plants on 1/2 MS, 0.1% agar powder liquid medium with 6% glucose. (B) Root length of seedlings under the condition of (A), 6-10 seedlings were measured for each transient assay.

**
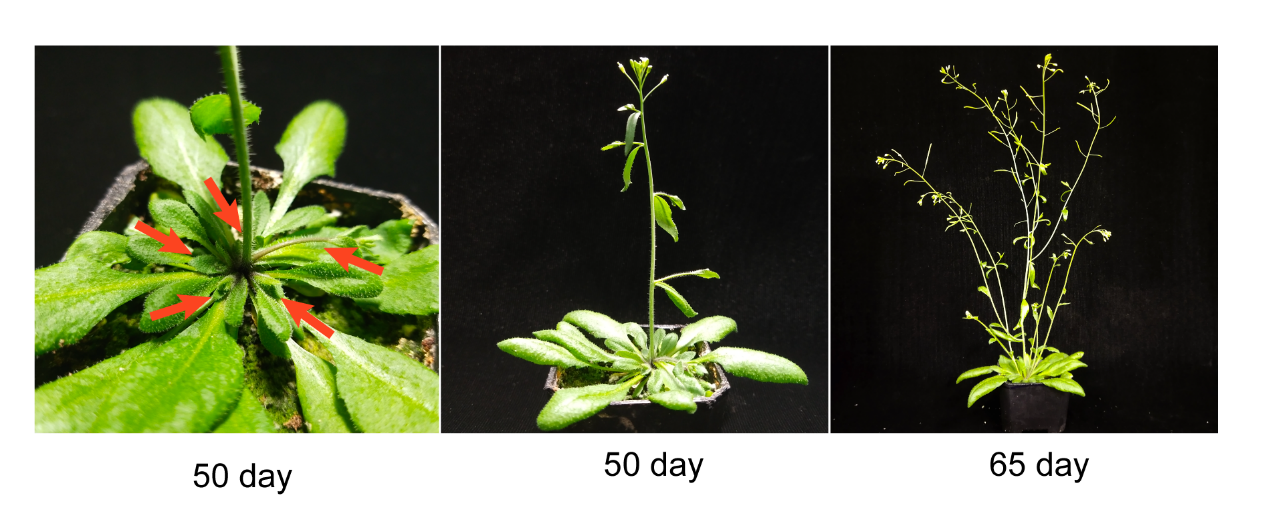
**

**Supplementary Figure 5** Plant phenotype of *35S::FpHXK1* transgenic Arabidopsis on 50^th^ or 65^th^ day.


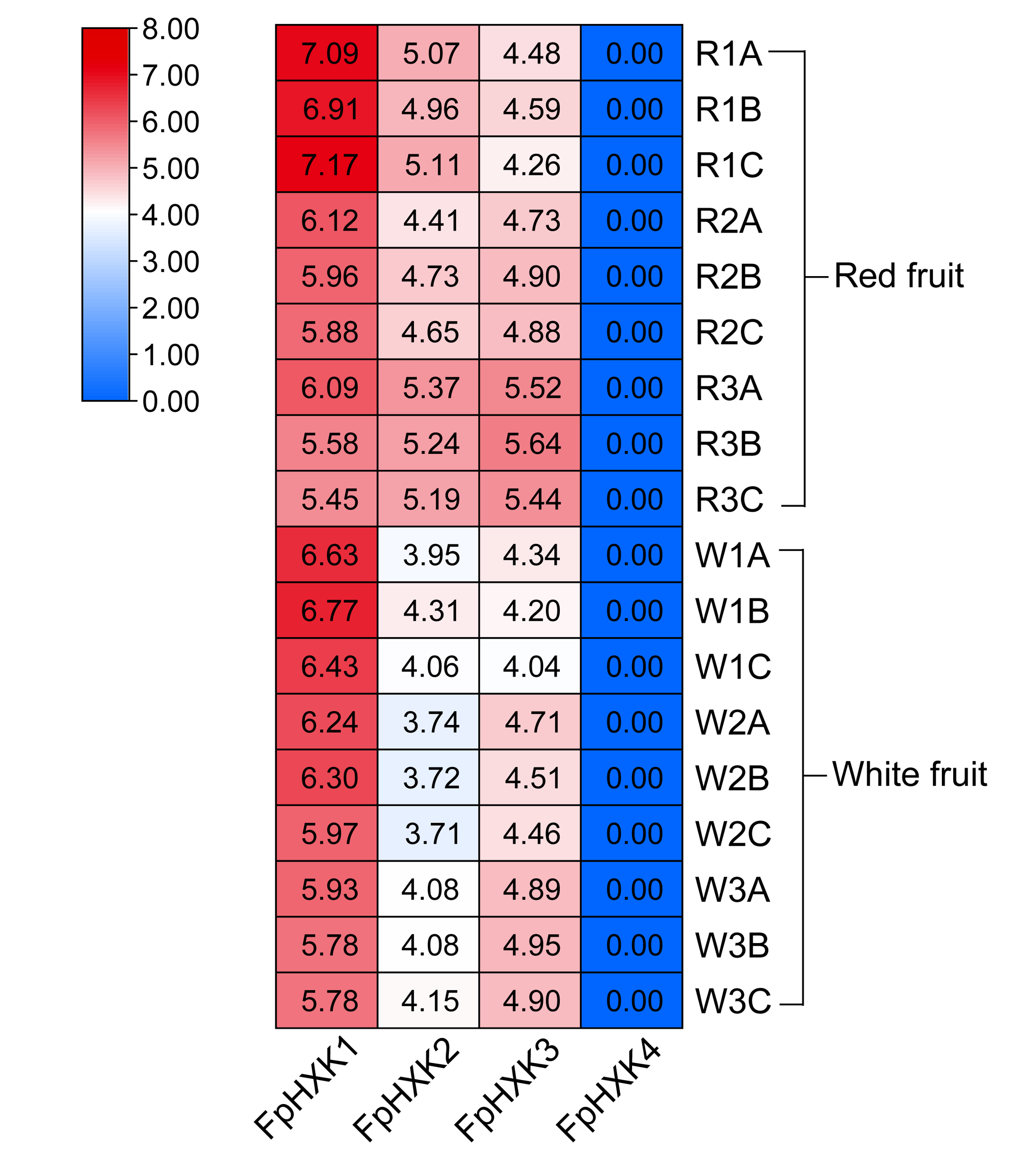


**Supplementary Figure 6** Heatmap of FpHXK1-4 gene expressions in red fruits and white fruits of *F. pentaphylla*. Ripe stages descriptions: R1A-C, green stage of the red fruit; R2A-C, turning stage of the red fruit; R3A-C, red ripping stage of the red fruit; W1A-C, green stage of the white fruit; W2A-C, turning stage of the white fruit; W3A-C, ripping stage of the white fruit.
